# Galactose-Clicked Curcumin-Mediated Reversal of Meropenem Resistance among *Klebsiella pneumoniae* by Targeting Its Carbapenemases and the AcrAB-TolC Efflux System

**DOI:** 10.3390/antibiotics10040388

**Published:** 2021-04-04

**Authors:** Shivangi Yadav, Ashish Kumar Singh, Anand K. Agrahari, Akhilesh Kumar Pandey, Munesh Kumar Gupta, Dipshikha Chakravortty, Vinod Kumar Tiwari, Pradyot Prakash

**Affiliations:** 1 Bacterial Biofilm and Drug Resistance Research Laboratory, Department of Microbiology, Institute of Medical Sciences, Banaras Hindu University, Varanasi 221005, India; yadavshivi2706@gmail.com (S.Y.); chunchunpratapsingh@gmail.com (A.K.S.); guptamuneshkumar@gmail.com (M.K.G.); 2 Department of Microbiology and Cell Biology, Indian Institute of Science, Bengaluru 560012, India; dipa@iisc.ac.in; 3 Department of Chemistry, Institute of Science, Banaras Hindu University, Varanasi 221005, India; anand32bhu@gmail.com; 4 Department of Biochemistry, Institute of Science, Banaras Hindu University, Varanasi 221005, India; akppdy@googlemail.com; 5 Center for Biosystem Science and Engineering, Indian Institute of Science, Bengaluru 560012, India

**Keywords:** bliss modelling, synergism, AcrAB-TolC, bla_KPC, mCIM, eCIM

## Abstract

In over eighty years, despite successive antibiotics discoveries, the rapid advent of multidrug resistance among bacterial pathogens has jolted our misapprehension of success over them. Resistance is spreading faster than the discovery of new antibiotics/antimicrobials. Therefore, the search for better antimicrobials/additives becomes prudent. A water-soluble curcumin derivative (Cur_aq_) was synthesised, employing a Cu (I) catalysed 1, 3-cyclo addition reaction; it has been evaluated as a potential treatment for multidrug-resistant isolates and as an antibiotic adjuvant for meropenem against hypervirulent multidrug-resistant *Klebsiella pneumoniae* isolates. We also investigated its solubility and effect over carbapenemase activity. Additionally, we investigated its impact on the AcrAB-TolC system. We found that Cur_aq_ inhibited bacterial growth at a minimal concentration of 16 µg/mL; at a 32 µg/mL concentration, it killed bacterial growth completely. Only nine (9.4%) *Klebsiella* isolates were sensitive to meropenem; however, after synergising with Cur_aq_ (8 µg/mL), 85 (88.54%) hvKP isolates became sensitive to the drug. The Cur_aq_ also inhibited the AcrAB-TolC efflux system at 1 µg/mL concentration by disrupting the membrane potential and causing depolarisation. The kinetic parameters obtained also indicated its promise as a carbapenemase inhibitor. These results suggest that Cur_aq_ can be an excellent drug candidate as a broad-spectrum antibacterial and anti-efflux agent.

## 1. Introduction

*Klebsiella pneumoniae*, a Gram-negative bacillus of the *Enterobacteriaceae* family, is an opportunistic pathogen noted for commonly infecting immunocompromised individuals [1]. *Klebsiella pneumoniae* is distributed far and wide, from soil to vegetation. It accounts for many healthcare-associated infections in humans, including pneumonia, wound/surgical site infections, septicemia, and meningitis. Additionally, it forms biofilms efficiently over medical devices (e.g., catheters and endotracheal tubes) [2]. Reports have noted an upsurge of phenotypically unique hypervirulent isolates of *Klebsiella pneumoniae* (hvKP), efficiently producing capsular polysaccharides [3]. These hvKP isolates are astoundingly invasive, adapted for infecting even healthy individuals. They are responsible for community-acquired infections, such as severe pneumonia, urinary tract infections, pyogenic liver abscess, and necrotising fasciitis. Exceptionally, these hvKP isolates are capable of disseminating to infect other organs as well, complicating treatment—unlike their classical counterparts. Moreover, K. *pneumoniae* have developed antimicrobial resistance, most recently to the virtually last resort of treatment: carbapenems [4,5]. Multidrug pumps (predominantly in the clinically relevant AcrAB-TolC and Mex pumps of the resistance-nodulation-division (RND) superfamily among *Klebsiella pneumoniae*) not only arbitrate intrinsic and acquired multidrug resistance (MDR); they are also implicated in stress response and pathogenicity. Furthermore, these efflux pumps are also reported to interact synergistically with other resistance mechanisms that further intensify resistance levels [6].

Among other *Enterobacteriaceae* members like *E. coli*, it has been ascertained that carbapenem-resistant isolates devoid of carbapenemase production present a noticeable reduction in intracellular antibiotic accumulation, owing to the overexpression of various efflux pumps and porin alteration [7]. Reports suggest that the reduced susceptibility of *K. pneumoniae* against both ertapenem and imipenem is attributed to the overexpression of resistance-nodulation-division (RND) family efflux genes [8]. However, against meropenem, the sensitivity remained unperturbed. In synergy with other resistance mechanisms (e.g., alterations in membrane permeability, enzyme-mediated inactivation and modification of antibiotics, target alteration), these resistant bacterial infections become almost impossible to treat [9]. *K. pneumoniae* produces several enzymes—oxacillinases, extended-spectrum β-lactamases, Metallo-β-lactamases, and carbapenemases—that can degrade various β-lactam antibiotics and carbapenems [5]. These features become more devastating in the current scenario due to the dwindling number of effective antibiotics. Therefore, it has been listed as one of the critical threats by the World Health Organization. The escalated carbapenem resistance among *K. pneumoniae* isolates witnessed in the last decade across the globe (and ostensible lack of in-depth study of carbapenem resistance, especially meropenem) reveals the dire necessity for baseline exploration [8]. Detailed knowledge about antimicrobial susceptibility patterns and carbapenemase production status is a prerequisite for managing and treating *K. pneumoniae* infections. Genetic diversity (in terms of clonal relatedness, for identifying the more prevalent strain) can help prevent and control infections in hospitals.

Considering the significant role of efflux pumps in arbitrating resistance in *Klebsiella pneumoniae*, targeting the bacterial efflux systems is very relevant. AcrAB-TolC efflux pump-mediated carbapenem resistance has been well documented against imipenem and ertapenem but was not reported to be significantly associated with meropenem resistance. In our present study, we observed the role of the efflux pump against meropenem. 

Further, in addition to contributing to the multidrug resistance phenotype, the AcrAB efflux pump may represent a novel virulence factor required for K. pneumoniae to resist innate immune defense mechanisms of the lung, thus facilitating the onset of pneumonia [10,11]. To the best of our knowledge, among *Klebsiella pneumoniae* isolates, the efflux pump’s role in acquiring meropenem resistance has not been elucidated.

Curcumin is one of the most studied polyphenols extracted from *Curcuma longa*’s rhizomes; literature is inundated with reports documenting its promise to target the complex features of multidrug resistance (MDR) [12]. However, the doses required are much higher due to phenolic rings and their β-diketone unit [13]. The major limitation of using curcumin as a treatment option is its high hydrophobicity and instability at elevated pH levels. Despite various attempts to improve its aqueous solubility and stability, the problem remains largely unanswered [14]. A more recent approach reported attempted use of nano and quantum formulations. These quantum dots’ biological activities have shown promise, but as it is the nano/quantum formulation, its systemic use is still debatable [15,16]. 

Addressing the urgent need for better antibacterial agents/antibiotic adjuvants, we herein reported a water-soluble bifunctional curcumin derivative and evaluated it’s potential to combat efflux-mediated resistance of meropenem. A significant number of meropenem-resistant isolates showed minimum inhibitory concentration (MIC) against meropenem in the sensitive range in the presence of a sub-inhibitory concentration of water-soluble curcumin.

This study aimed to report water-soluble bifunctional curcumin as a biocompatible efflux-pump inhibitor, carbapenemase inhibitor and meropenem-potentiating possible drug candidate, outlining the mechanistic underpinnings.

## 2. Results

### 2.1. Antimicrobial Susceptibility Profile

One hundred thirty-one isolates (131, 83.44%) of *K. pneumoniae* were multidrug-resistant as per our set criteria (Supplementary Figure S5). A majority of isolates (*n* = 155, 98.72%) were resistant to the aminopenicillin class (ampicillin). Similarly, 144 (91.72%), 154 (98.08%), and 138 (87.89%) isolates were found resistant to cephalosporins (namely cefepime, cefuroxime, and ceftazidime, respectively). Worryingly, a very similar trend was noted against carbapenem antibiotics. A total of 98 (62.42%), 115 (73.25%), and 111 (70.70%) isolates were found resistant to ertapenem, meropenem, and imipenem, respectively.

### 2.2. Detection of Carbapenemase Production

**Figure 1:**
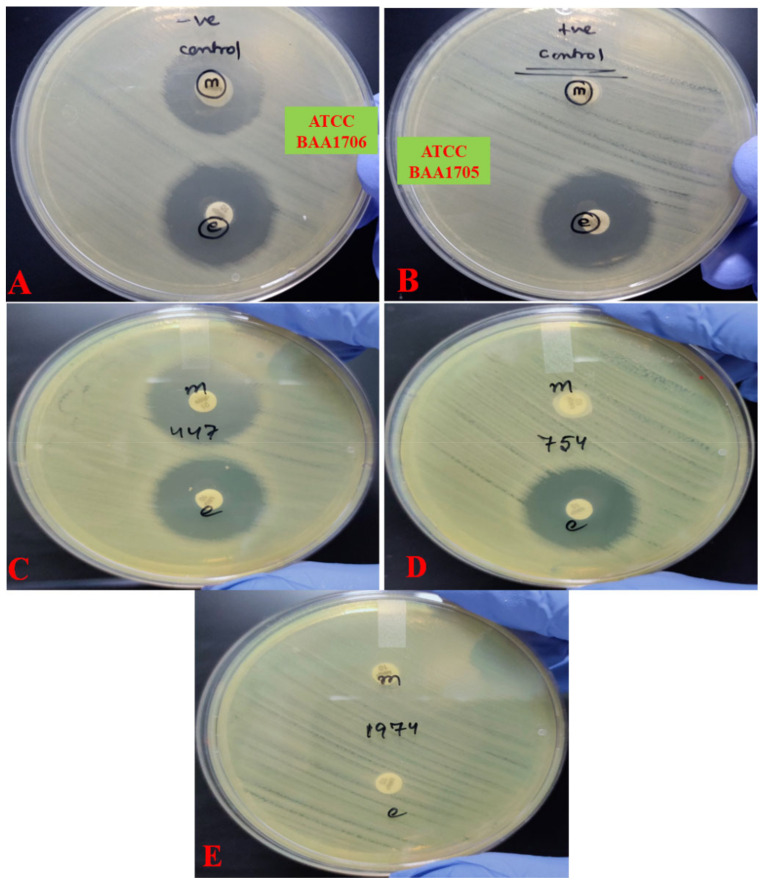
Pictorial demonstration of modified carbapenem inactivation method (mCIM), EDTA-carbapenem inactivation method (eCIM). (**A**) A negative control, where both mCIM and eCIM results are negative (ATCC BAA1706). (**B**) Positive control, where both mCIM and eCIM results are positive (ATCC BAA1705). (**C**) Representative clinical isolate (no-447) where both mCIM and eCIM results are negative. (**D**) Representative clinical isolate (no-754) where both mCIM and eCIM results are positive. (**E**) Representative clinical isolate (no-1974) where mCIM assay is positive while eCIM result is negative.

Out of 157 clinical *Klebsiella pneumoniae* isolates, 31 isolates (19.51%) were found positive for carbapenemase production by mCIM, while 65 isolates (41.40%) were found to be eCIM positive. Of note, a total of 61 isolates (38.85%) were found to be mCIM negative, indicating other mechanisms of resistance against meropenem, imipenem, and ertapenem (Table 1, Supplementary File S3). Interestingly, in the current study, we did not find any isolate exhibiting indeterminate results. Figure 1a,b represents, respectively, the phenotypic view of mCIM negative (ATCC BAA1706) and mCIM positive (ATCC BAA1705) control strains (Figure 1). Figure 1c–e represents test strains with three different results (Figure 1). 

### 2.3. Molecular Typing of Klebsiella Pneumoniae Isolates Using Enterobacterial Repetitive Intergenic Consensus (ERIC)-PCR

The ERIC-PCR profiles demonstrated that many isolates were genetically diverse, e.g., isolates 1, 3, and 12. However, some isolates (e.g., 7, 10, 30, 31, 79, and 82) were closely related. The phylogenetic tree (dendrogram) and the diced gel lanes generated after ERIC-PCR (Supplementary Figure S6) indicated significant intraspecific variations. The 96 clinical isolates that were found to be carbapenemase producers seemed to form 79 clusters. Each cluster depicted a different ERIC profile, describing the genetic diversity of *Klebsiella pneumoniae* clinical isolates (Supplementary Figure S6). 

### 2.4. Synthesis of Water-Soluble Curcumin (Cur_aq_)

The synthesis of galactose-clicked curcumin was accomplished as delineated earlier [17]. Briefly: initially, curcumin was propargylated by treating it with propargyl bromide in the presence of K_2_CO_3_ in dry DMF under an argon atmosphere at room temperature for 48 h. This yielded curcumin di-alkyne, as orange-red colored semisolid, in good yield (55%). After the synthesis of the alkyne part, a clickable azide part was constructed from D-galactose by implementing high yielding steps, including acetate protection (using acetic anhydride), followed by selective anomeric bromination (2,3,4,6-Tetra-O-acetyl-d-galactopyranosyl bromide). We then substituted, employing sodium azide in DMF to afford the galactose azide (2) in overall 74% yield. Furthermore, galactose azide (2) was reacted with curcumin di-alkyne (4) using CuSO_4_·5H_2_O and NaAsc in the presence of THF/H_2_O, which resulted in regioselective 1, 4 triazolyl product (**5**) in good yield. In the end, the synthesised scaffold 5 was deacetylated using the (Zemplèn reaction)-a procedure to obtain a free hydroxyl group of curcumin 1,4 triazolyl di-galactose (6). (For detailed experimentation, see the Results section). All reactions were performed at room temperature under standard conditions. Thin-layer chromatography (TLC) was executed on Merck 60 F_254_ silica gel, precoated on aluminum plates and exposed under a UV lamp (λ_max_ @ 254 nm) by spraying with the methanolic-H_2_SO_4_ solution. This was followed by charring (for carbohydrate moieties) by heating at ~100 °C–120 °C. NMR spectra were recorded on a JEOL AL500 FT-NMR spectrometer (500 MHz for ^1^H NMR and 125 MHz for ^13^C NMR) in CDCl_3,_ DMSO, and D_2_O (for deacetylated scaffold). Chemical shifts were provided in ppm downfield from internal *TMS,* and *J* values were depicted in Hz. Column chromatography was performed using silica gel 234–400 mesh, E-Merck grade using n-hexane, ethyl acetate and DCM as the eluent.

### 2.5. Photophysical Behaviour and Solubility

**Figure 2:**
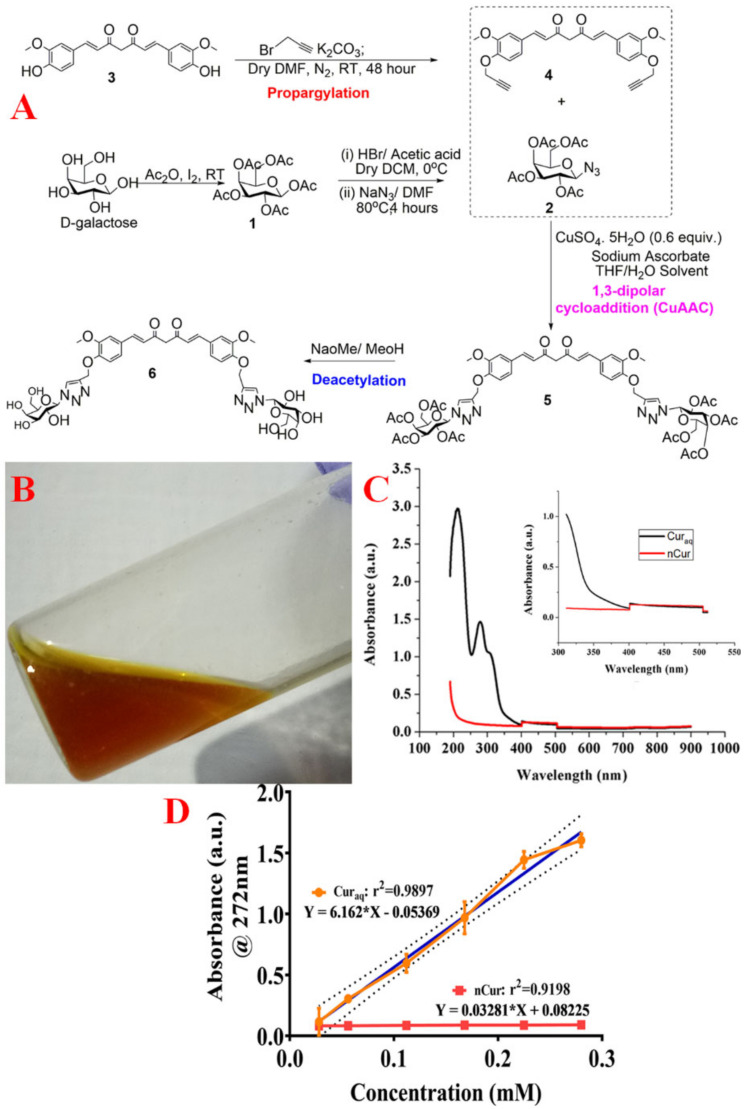
(**A**) Scheme for the synthesis of water-soluble curcumin (Cur_aq_). (**B**) Physical appearance of Cur_aq_. (**C**) UV-Vis spectrum of Cur_aq_ and native curcumin (nCur). Inset is the zoomed-in view of the spectrum from 300 nm to 550 nm. (**D**) Standard plots of absorbance v/s concentration for Cur_aq_ and nCur at 272 nm.

At first, we dissolved the Cur_aq_ (10 mg/mL) in MilliQ water to see its physical appearance (Figure 2b). Unlike the aqueous insolubility of native curcumin (perceived as yellow floccules present at the top of the aqueous phase and heterogeneity of suspension, seen throughout, as reported earlier) we noted a dark yellow (mustard oil) color in the water-soluble curcumin derivative [12].

The UV-Visible absorption spectrum of Cur_aq_ was recorded in water (Figure 2c). Three absorption bands were obtained for Cur_aq_ at ~192, 272, and 324 nm. The absorption bands at ~192 and 272 nm were obtained in the UV region of the electromagnetic spectrum. In contrast, a broad absorption band was obtained at ~425–512 nm region and a shoulder at ~324 nm extending to the visible region (Figure 2c).

Finally, we compared the solubility of Cur_aq_ with native curcumin. We plotted the calibration curve by plotting the concentrations versus absorbance at λ_max_ ~272 nm, and then the slope was calculated from the plot (~0.2359 for Cur_aq_ slope, ~0.5409 for native curcumin slope). We then dissolved the maximum dissolvable amount of Cur_aq_ into MilliQ water and removed the undissolved part by centrifugation. At the same λ_max,_ we determined the absorbance of unperturbed MilliQ and native curcumin. We then calculated the solubility by applying the following Equation (1):

(1) $$Solubility=\left(Absorbanceat\lambda max\right)/Slope$$

The solubility of Cur_aq_ was 32,146.91 times higher than its native form (soluble up to 5.934 mg/mL, while native curcumin is soluble up to 0.185 µg/mL).

### 2.6. Minimum Inhibitory Concentration (MIC) Determination among Carbapenemase Producers

#### 2.6.1. Minimum Inhibitory Concentration (MIC) Determination

The MIC of meropenem for 7 (7.3%) out of the 96 tested clinical isolates was less than 1 µg/mL (Table 2). Only 9 (9.4%) out of 96 clinical isolates were found to be sensitive to meropenem. However, among the carbapenem class, the sensitivity profile of ertapenem (*n* = 13, 13.54%) was better than that of meropenem, which in turn was followed by imipenem (*n* = 11, 11.45%) (Table 2). Among the protonophores, valinomycin was highly effective with a MIC value ranging from 1–64 µg/mL. Similarly, the most ineffective was verapamil, whose MIC ranged from 32–512 µg/mL. However, the MIC of CCCP ranged from 16–32 µg/mL. Similarly, the MIC of Cur_aq_ ranged from 16–64 µg/mL (Supplementary Figure S4). 

#### 2.6.2. Alteration in Minimum Inhibitory Concentration (MIC) after Adding CCCP, Verapamil, Valinomycin and Cur_aq_

The effect of CCCP, verapamil, valinomycin and Cur_aq_ on the growth of drug-resistant *Klebsiella* isolates combined with meropenem was investigated in microtiter plates by the log_10_ CFU/mL reduction, and a tally of fold reduction is represented in Table 3. However, the MIC of meropenem was found to range between 4–256 µg/mL (*n* = 87) against resistant isolates of *K. pneumoniae*; after adding Cur_aq_, the MIC was reduced significantly to between ≤1–8 µg/mL. Earlier, only 9 (9.4%) *Klebsiella* isolates were found to be sensitive to meropenem. After adding Cur_aq_ (8 µg/mL), 85 (88.54%) isolates became sensitive to meropenem. However, native curcumin failed to potentiate meropenem in the tested concentration range. Similarly, 59 (61.45%), 36 (37.5%), and 74 (77.08%) isolates became sensitive to meropenem after adding CCCP, verapamil, and valinomycin, respectively (Table 4).

#### 2.6.3. Phenotypic Observation of Synergy and Its Quantification Using the Bliss Model

**Figure 3:**
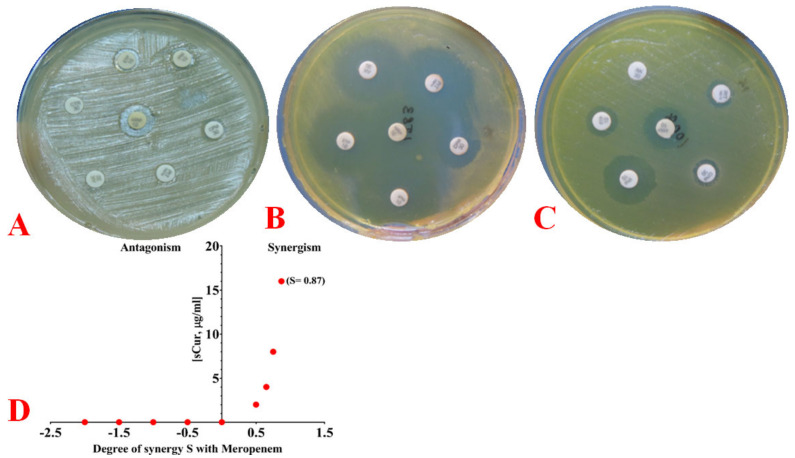
(**A**) Phenotypic depiction of resistance (employing Kirby–Bauer disc diffusion method) of a *Klebsiella pneumoniae* strain against 7 drugs, namely: imipenem (7 mm), meropenem (11 mm), ertapenem (8 mm), cefepime (5 mm), piperacillin-tazobactam (5 mm), amikacin (5 mm), and ceftriaxone (5 mm). (**B**) Phenotypic depiction of resistance (employing Kirby–Bauer disc diffusion method) of a *Klebsiella pneumoniae* strain against 6 drugs, namely: imipenem (28 mm), meropenem (29 mm), ertapenem (28 mm), cefepime (16 mm), piperacillin-tazobactam (27 mm), and amikacin (26 mm) after adding 8 µg/mL Cur_aq_ in the agar medium. (**C**) Phenotypic depiction of resistance (employing Kirby–Bauer disc diffusion method) of a *Klebsiella pneumoniae* strain against 6 drugs, namely: imipenem (21 mm), meropenem (18 mm), ertapenem (10 mm), cefepime (8 mm), piperacillin-tazobactam (7 mm), and amikacin (5 mm) after adding 16 µg/mL nCur in the agar medium. (**D**) The Bliss Model for synergy, confirming a synergistic effect (S = 0.87), between Cur_aq_ and meropenem against *Klebsiella pneumoniae*.

The significant reduction in MIC of meropenem upon addition with Cur_aq_ indicated its potential synergism. Therefore, at first, it was observed over a solid medium by dissolving the sub-inhibitory concentrations of native curcumin and Cur_aq_ in the agar mediums. Then, a change in the zone of inhibition was observed against in-vogue drugs, particularly meropenem. We noted a significant increase in the zone of inhibitions of all in-vogue drugs over the plate with Cur_aq_ compared to untreated ones (Figure 3a,b). However, native curcumin failed to show such significant alterations (Figure 3c). After phenotypic observations, the possible synergism of meropenem with Cur_aq_ was then quantified using the Bliss model of synergy. The degree of synergism was found to be 0.87, which indicated significant synergy between meropenem and Cur_aq_ at all investigated concentrations (Figure 3d). 

Subsequently, we determined the FIC index as 0.15, which endorsed the potential synergism.

### 2.7. Detection of Multidrug Resistance and Virulence Determinants Using PCR

**Figure 4:**
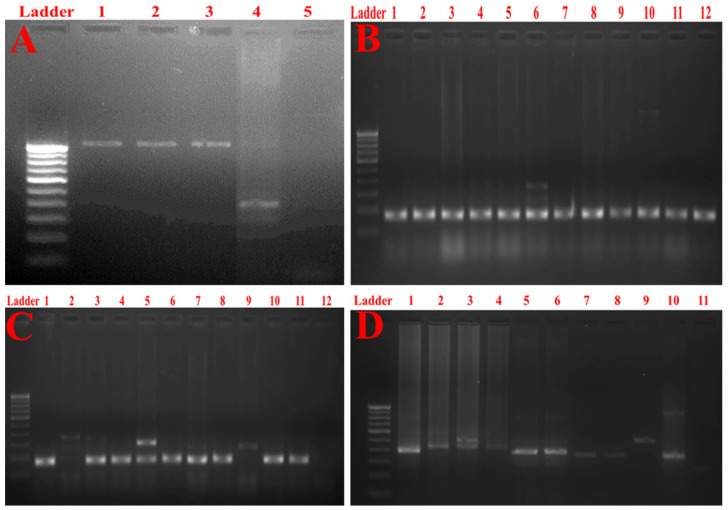
(**A**) Gel image of the PCR for bla__KPC_ gene detection. Lane 1: bla__KPC_ gene present (size 1010 bp) in the positive control. Lane 2: bla__KPC_ gene present (size 1010 bp) in representative isolate 754. Lane 3: bla__KPC_ gene present (size 1010 bp) in representative isolate 1974. Lane 4: bla__KPC_ gene absent (size 1010 bp) in representative isolate 447. Lane 5: bla__KPC_ gene absent (size 1010 bp) in the negative control. (**B**) Gel image of the PCR product for FimH-1 detection among blood and pus isolates. Lane 1: FimH-1 gene (size 180 bp) present in the positive control. Lanes 2–12: FimH-1 gene (size 180 bp) present in the test blood (lanes 2–7) and pus (lanes 8–12) isolates. (**C**) Gel image of the PCR product for FimH-1 detection among urine isolates. Lane 1: FimH-1 gene (size 180 bp) present in the positive control. Lanes 2–12: FimH-1 gene (size 180 bp) present in the test urine isolates. Note the absence of FimH-1 bands in lanes 2, 9 and 12. (**D**) Gel image of the PCR product for RmpA gene detection. Lane 1: RmpA gene (size 435 bp) present in the positive control. Lanes 2–10: RmpA gene (size 435 bp) present in the test isolates. Lane 11: RmpA gene (size 435 bp) absent in the negative control.

Figure 4A depicts the gel image of bla__KPC_ gene detection. The results obtained by mCIM and eCIM phenotypic assays were endorsed by genetic determination by detecting the bla__KPC_ gene. The FimH-1 gene coding for type 1 and type 3 fimbrial adhesins was found in all pus and blood isolates (Figure 4B). However, FimH-1 was found less prevalently among urine isolates (Figure 4C). Similarly, all the tested *Klebsiella* isolates, irrespective of their source of isolation, tested positive for the presence of the RmpA gene, responsible for capsular polysaccharide synthesis (Figure 4D). It is worth mentioning that virulence profiles were found to vary among *K. pneumoniae* strains that possessed the same clonal relationship by ERIC-PCR.

### 2.8. Detection of Major Efflux Pumps System

**Figure 5:**
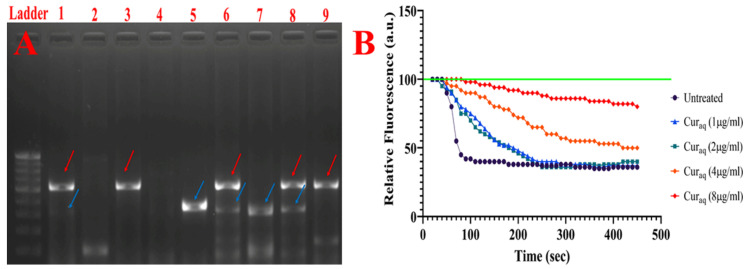
(**A**) Gel image of the PCR product for AcrAB-TolC efflux pump system detection. Lane 1: TolC gene (size 527 bp) present in the positive control (red arrow). Lane 2: Neither TolC nor AcrAB genes present in the negative control. Lane 3: TolC gene present (size 527 bp) in the test isolate (red arrow). Lane 4: Neither TolC nor AcrAB genes present in the test isolate. Lane 5: AcrAB gene (size 312 bp) present in the positive control (blue arrow). Lane 6: Both TolC (red arrow) and AcrAB (blue arrow) genes present in the test isolate. Lane 7: AcrAB gene (size 312 bp, blue arrow) present in the test isolate. Lane 8: Both TolC (red arrow) and AcrAB genes (blue arrow) present in the test isolate. Lane 9: TolC gene present (size 527 bp, red arrow) in the test isolate. (**B**) Dose-dependent inhibition of 1,2’ DNA efflux by Cur_aq_ in the *Klebsiella pneumoniae*. Energization initiated with 50 mM glucose at 20 s. Pre-energization fluorescence intensity adjusted to 100 relative fluorescence units (RFU).

The high prevalence of the multidrug efflux pump system (AcrAB-TolC) was significantly correlated with the MDR pattern in our study. The AcrAB-TolC efflux pump system was present among 87.62% of the total tested ICU isolates of *Klebsiella pneumoniae*. However, among 12 isolates, an incomplete AcrAB-TolC efflux pump system was detected, signifying that TolC was not present (Figure 5a).

### 2.9. 1,2′-Dinaphthylamine Accumulation Assay

The current study found that both Cur_aq_ and CCCP inhibited 1,2’ DNA efflux in a dose-dependent manner (Figure 5b). The first efflux inhibitory effects of Cur_aq_ were seen at 1 µg/mL, whereas Cur_aq_ abolished the efflux almost entirely at 8 µg/mL. Efflux inhibition at 2 µg/mL concentration was not significantly higher than that at 1 µg/mL. 

### 2.10. Expression Pattern of acrA, acrB and TolC in the Presence of Cur_aq_

**Figure 6:**
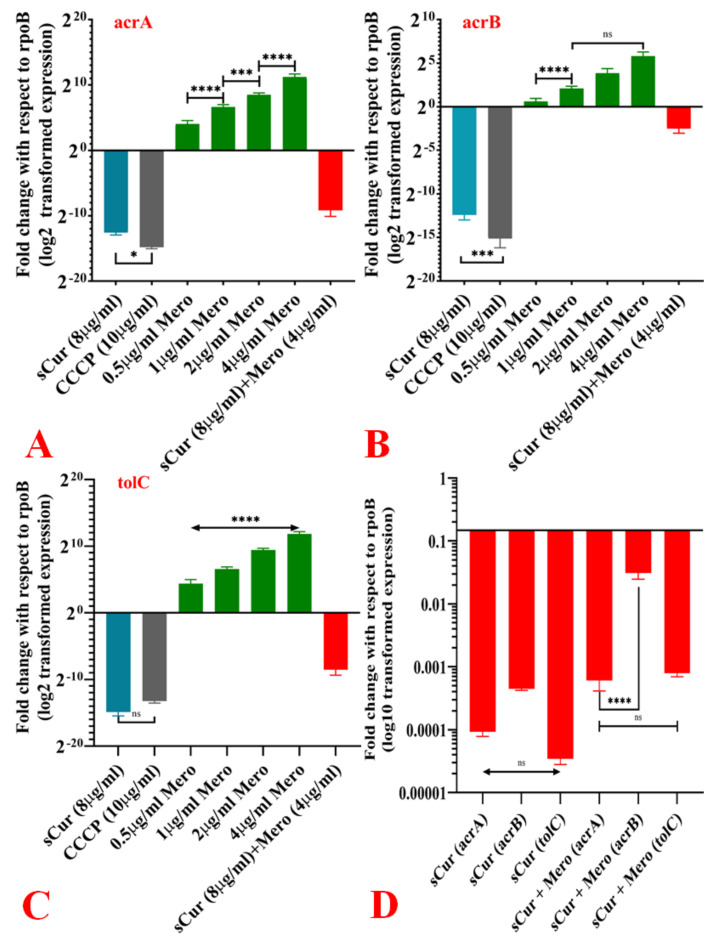
Effect of different variables on the expression profile of the AcrAB-TolC system. (**A**) Effect of Cur_aq_ (8 µg/mL), CCCP (10 µg/mL), various concentrations of meropenem (0.5–4 µg/mL), and meropenem (4 µg/mL) with Cur_aq_ (8 µg/mL) on the expression of acrA. (**B**) Effect of Cur_aq_ (8 µg/mL), CCCP (10 µg/mL), various concentrations of meropenem (0.5–4 µg/mL), and meropenem (4 µg/mL) with Cur_aq_ (8 µg/mL) on the expression of acrB. (**C**) Effect of Cur_aq_ (8 µg/mL), CCCP (10 µg/mL), various concentrations of meropenem (0.5–4 µg/mL), and meropenem (4 µg/mL) with Cur_aq_ (8 µg/mL) on the expression of tolC. (**D**) Effect of Cur_aq_ (8 µg/mL) and combinations of meropenem (4 µg/mL) with Cur_aq_ (8 µg/mL) on the expression of acrA, acrB and tolC.

Meropenem-induced over-expression of the AcrAB-TolC efflux system was noted among eighty-four isolates (86.6%); however, the differential expressions of acrA, acrB, and tolC in the presence of different concentrations of meropenem were found to be changed. For instance, meropenem triggered the over-expression of acrA in a dose-dependent manner. At all the tested concentrations, meropenem significantly triggered the expression of acrA; however, at 1 and 4 µg/mL concentration, the augmentation was more significant (Figure 6a). The synthesised Cur_aq_ significantly downregulated the meropenem-induced overexpression of acrA (Figure 6a). However, the expression of acrB was different. It remained almost the same at all tested concentrations except for 0.5 and 1 µg/mL. (Figure 6b). The Cur_aq_ was found to downregulate the expression of acrB; however, compared to acrA, downregulation was not very pronounced. Interestingly, the transcriptional expression of tolC was significantly augmented in response to the increasing concentration of meropenem (Figure 6c). Similarly, to the case of acrA, Cur_aq_ significantly inhibited the expression of tolC. Overall, the synthesised Cur_aq_ was found to dramatically downregulate the tripartite AcrAB-TolC efflux system, except for acrB (in comparison to acrA and tolC). However, it was found to be significantly downregulated in comparison to the meropenem-induced expression (Figure 6d).

### 2.11. Effect over Carbapenemases

#### 2.11.1. Spectrophotometric Analysis of Meropenem

**Figure 7:**
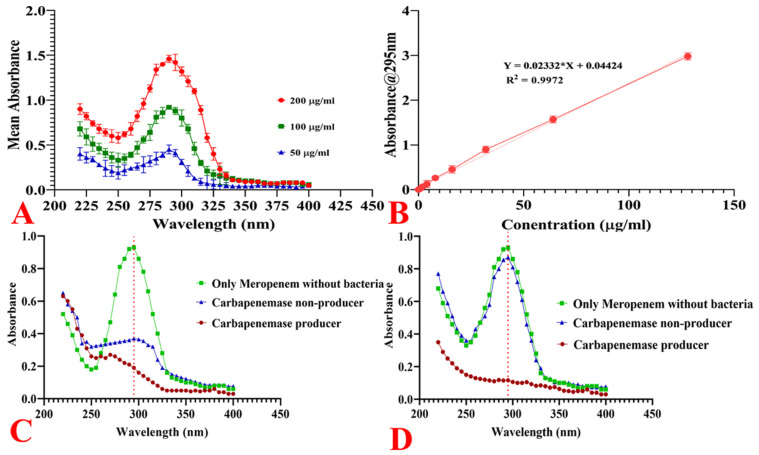
(**A**) Absorption spectra of meropenem at 50, 100, and 200 µg/mL concentrations. The bars show standard deviations of three independent replicates. (**B**) Relationship between the meropenem concentration and the absorbance at 295 nm. Curve-fitting with linear regression analysis performed (R^2^ = 0.9972). (**C**) Spectral analysis of meropenem hydrolysis before background correction. (**D**) Spectral analysis of meropenem hydrolysis after background correction.

The absorption spectrum of meropenem in phosphate-buffered saline (PBS) was examined within the wavelength range of 220 nm to 400 nm. We noted the absorption peak at 295 nm (Figure 7a). The absorbance of meropenem at 295 nm correlated linearly with concentrations of up to 128 μg/mL (Figure 7b). 

#### 2.11.2. Hydrolysis of Meropenem by Bacterial Cells

Enzymatic hydrolysis of meropenem by carbapenemase-producing and non-carbapenemase-producing strains was examined by spectrophotometry. The direct comparison of the gross absorbance of carbapenemase-producing and non-producing bacterial suspensions with meropenem was cumbersome at 295 nm, owing to differences in the baseline absorbance spectra of each bacterial type (Figure 7c). Therefore, we performed baseline correction by subtracting a bacterial suspension’s absorbance without meropenem from the meropenem containing the bacterial suspension. Carbapenemase-producing bacteria-hydrolysed meropenem was evident from the disappearance of the absorption peak of meropenem at 295 nm. In contrast, the meropenem peak remained almost intact in non-carbapenemase-producing bacterial suspension (Figure 7d). 

#### 2.11.3. Quantification of Bacterial Hydrolytic Activity to Meropenem

**Figure 8:**
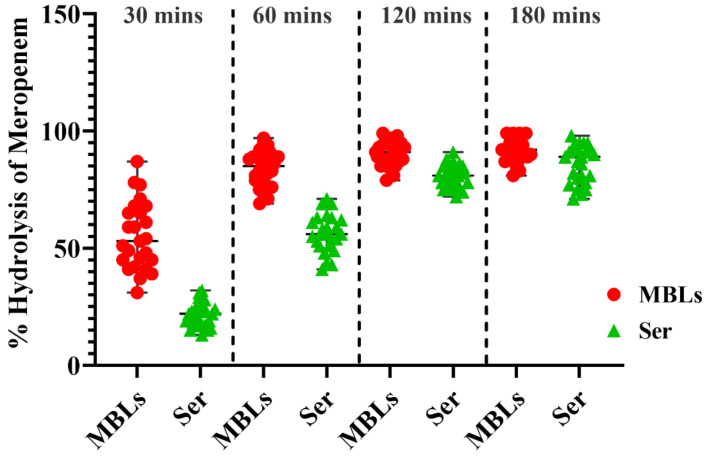
The time-dependent % hydrolysis of meropenem by Metallo β-lactamase (MBLs)-producing *Klebsiella pneumoniae* (*n* = 25) and serine carbapenemase-producing *Klebsiella pneumoniae* (*n* = 25) isolates after 30, 60, 120, and 180 min of incubation. The black bars show the median with the interquartile range. The horizontal dashed lines show the separation zones for each time.

The hydrolytic activities of clinical test isolates were investigated using spectrophotometry, and % hydrolysis was calculated. Metallo β-lactamase-type carbapenemase producers (both mCIM and eCIM positive, *n* = 25) exhibited significantly higher hydrolytic activities compared to serine-type carbapenemases (mCIM positive but eCIM negative, *n* = 25) after a 30-min incubation with meropenem (Figure 8). With increasing incubation times, % hydrolysis by both types of carbapenemase producers gradually increased (Figure 8). However, after 120 min of incubation, no significant difference in % hydrolysis was noted among isolates with different MICs (Figure 8). 

#### 2.11.4. Carbapenemases Inhibition Kinetics by Cur_aq_

In the presence of Curaq, the carbapenemase inhibition was found nearly linear at all time points. The initial reaction rate for each substrate or substrate-inhibitor concentration under steady-state conditions (<5% of substrate consumed) was calculated from the slope of the initial linear phase of the respective time course. Inhibition constants (*K_i_*) were evaluated by nonlinear fitting of the initial velocities at various concentrations of the substrates and inhibitors. The equations for different inhibition models were implemented in GraphPad 8.3.0. Best fits were obtained with the noncompetitive inhibition model using the Equation (2):

(2) $$v=\;\frac{V_{max}\;\left[S\right]}{\left(K_{m}+\left[S\right]\right)\left(1+\frac{\left[I\right]}{K_{i}}\right)}$$

**Figure 9:**
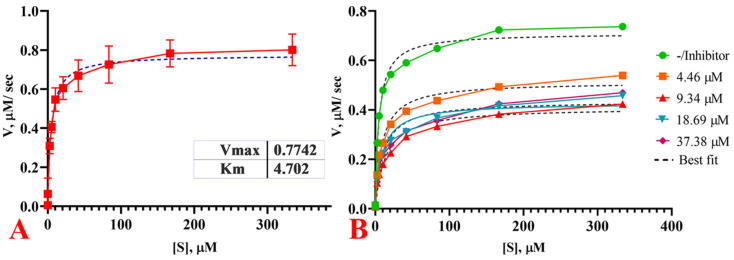
(**A**) Michaelis–Menten (MM) plot of the hydrolysis of meropenem by carbapenemase crude extract for 15 min. (**B**) MM plot of inhibition of carbapenemase-mediated meropenem hydrolysis by Cur_aq_ in crude extract for 15 min.

The inhibition constants (*K_i_*) and % inhibitions are shown with their corresponding standard errors obtained from the fit (Table 5). The reaction progress curves reflected a single unperturbed velocity (at each concentration) for the onset and inhibition progression (Figure 9). However, the addition of Cur_aq_ at 8 µg/mL concentration inhibited meropenem hydrolysis by 50% in tested *Klebsiella* isolates. At higher doses of 16 and 32 µg/mL, inhibition was relatively weaker (44.86% and 44.96%, respectively). The somewhat lessened hydrolysis inhibition may have been due to differences in cellular permeability based on the concentration gradient. This needs further elucidation.

### 2.12. Measurement of the Electrical Potential

**Figure 10:**
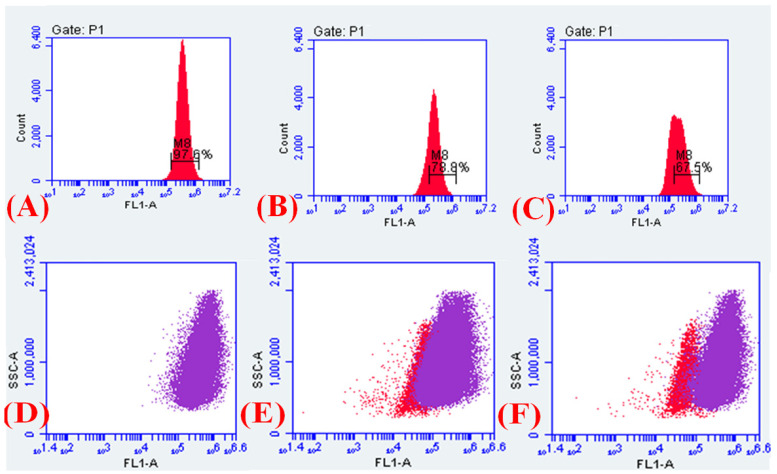
Flow cytometry analyses of *Klebsiella pneumoniae* with DiOC_2_(3) to explore changes in its electrical potential, unveiling the progressive leftward shifting indicative of alterations in electric potential. In total, 1,000,000 cells taken into account for each analysis. (**A**) Histogram of untreated logarithmic *Klebsiella* cells labeled with DiOC_2_(3). Note that most of the cell population (97.6%) retain the dye, indicating unperturbed membrane electric potential. (**B**) Histogram of Cur_aq_ (8 µg/mL)-treated *Klebsiella* isolates labeled with DiOC_2_(3). Note the significant left-shifting (78.8%) and decrease in intensity, indicating perturbations in membrane electrical potential. (**C**) Histogram of Cur_aq_ (8 µg/mL) and meropenem (4 µg/mL) combination-treated *Klebsiella* isolates labeled with DiOC_2_(3). Note the further left-shifting of the treated cell population (67.5%) and a considerable decrease in intensity compared to the untreated population, indicating significant perturbations in the membrane electrical potential. (**D**) Dot plot of untreated logarithmic *Klebsiella pneumoniae* cells labeled with DiOC_2_(3). (**E**) The overlay of the dot plot of Cur_aq_ (8 µg/mL)-treated *Klebsiella pneumoniae* tagged with DiOC_2_(3). Note the left-shifting (78.8%) of the red dots from the bulk purple dots, indicating perturbations in membrane electrical potential. (**F**) The overlay of the dot plot of Cur_aq_ (8 µg/mL) and meropenem (4 µg/mL) combination-treated *Klebsiella* isolates labeled with DiOC_2_(3). Note the left-shifting (67.5%) of the red dots from the bulk purple dots indicating augmented perturbations in membrane electrical potential fostered by the combination.

The flow cytometry and confocal microscopic studies revealed Cur_aq_-mediated perturbations in membrane electric potential using DiOC_2_(3)-based assay. As depicted in Figure 10, we confirmed that Cur_aq_ and meropenem in combination (8 µg/mL and 4 µg/mL) fostered considerable back-shift in the DiOC_2_(3) fluorescence—and hence augmented left shifting (Figure 10). This was analogous to the known electric potential disrupter CCCP. However, the incorporation of native curcumin, even at a concentration of 512 µg/mL, resulted only in slight left shifting, indicating moderately perturbed electric potential (Figure 10). The results suggest that the membrane potential of the Gram-negative *Klebsiella pneumoniae* was significantly altered after exposure to Cur_aq_ and meropenem. For area selection (in %) see Supplementary Information S7. 

**Figure 11:**
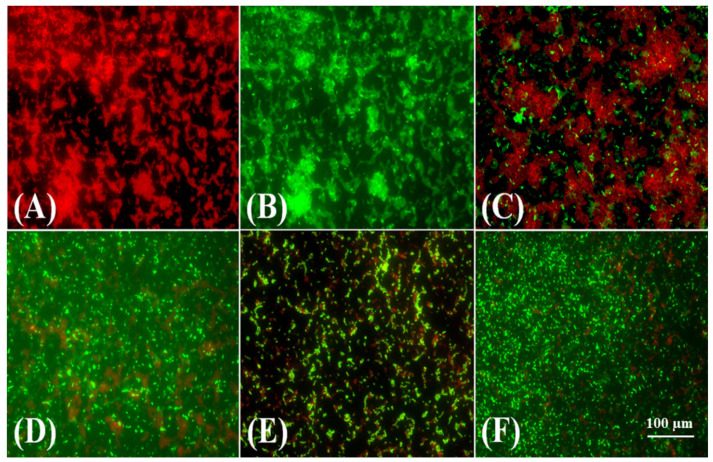
The confocal microscopic validation of electric potential perturbations mediated by meropenem (4 µg/mL), Cur_aq_ (8 µg/mL), and the combinations of Cur_aq_ (8 µg/mL) and meropenem (2, 4 µg/mL) over multidrug-resistant clinical *Klebsiella pneumoniae*. (**A**) Untreated *Klebsiella pneumoniae* at t = 0 h. (**B**) CCCP-treated *Klebsiella pneumoniae* at t = 18 h. (**C**) Meropenem (4 µg/mL)-treated *Klebsiella pneumoniae* at t = 18 h. (**D**) Cur_aq_ (8 µg/mL)-treated *Klebsiella pneumoniae* at t = 18 h. (**E**) Cur_aq_ (8 µg/mL)- and meropenem (2 µg/mL)-treated *Klebsiella pneumoniae* at t = 18 h. (**F**) Cur_aq_ (8 µg/mL)- and meropenem (4 µg/mL)-treated *Klebsiella pneumoniae* at t = 18 h.

The disruption of electric potential orchestrated by the Cur_aq_ and meropenem on multidrug-resistant clinical *Klebsiella pneumoniae* isolate (10825/2019) was further investigated by confocal microscopy. The freshly grown bacteria were then challenged with a sub-lethal concentration of Cur_aq_ (8 µg/mL) and meropenem (0.5, 1, 2, and 4 µg/mL), followed by staining with DiOC_2_(3) and subsequent imaging. Panel A depicts the untreated *Klebsiella pneumoniae* at the start time, while panel B is the CCCP-treated *Klebsiella pneumoniae* after 18 h of growth (Figure 11A,B). One can note intense green signals coming out, specifying the astounding CCCP-mediated membrane electrical potential perturbations. Interestingly, panel C represents the meropenem (4 µg/mL)-treated *Klebsiella pneumoniae* after 18 h, showing some green signals—indicating some alterations in the electrical potential (Figure 11C).

Interestingly, the test *Klebsiella* isolates exhibited a significant increase in green signals after treatment with Cur_aq_ (8 µg/mL), indicating the disruption of the bacterial cells’ electric potential (Figure 11D). Of note, the combination of Cur_aq_ (8 µg/mL) and meropenem (2 µg/mL) triggered a substantial increase in green signals, showing their relatively strong capacity to affect electric potential (Figure 11E). However, when the meropenem concentration was escalated to 4 µg/mL, extensive disruption in electric potential was perceived, as evinced by the intense green signals and diminished red signals (Figure 11). The results portray a dose-dependent impact on electrical potential through the combination of Cur_aq_ and meropenem.

### 2.13. Membrane Depolarisation Assay

**Figure 12:**
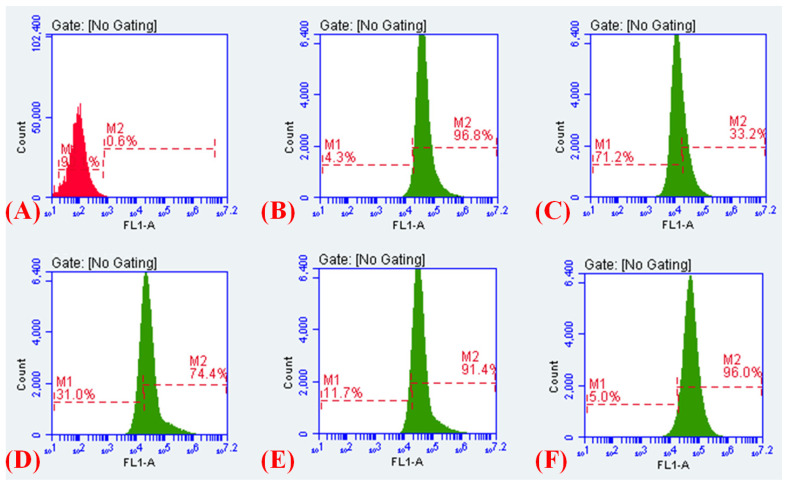
Flow cytometry analysis of *Klebsiella pneumoniae* with membrane potential-sensitive dye (DiSC_3_-5) to explore its membrane potential alterations. (**A**) Histogram of untreated *Klebsiella pneumoniae* cells labeled with DiSC_3_-5 (negative control). (**B**) Histogram of CCCP (10 µg/mL)-treated *Klebsiella pneumoniae* cells labeled with DiSC_3_-5 (positive control). The panels show the increase in cell population (96.8%) that took up DiSC_3_-5, depicting almost complete membrane depolarisation. (**C**) Histogram of meropenem (4 µg/mL)-treated *Klebsiella pneumoniae* cells labeled with DiSC_3_-5. The panels show the increase in the cell population (33.2%) that took up DiSC_3_-5, denoting the increase in membrane depolarisation. (**D**) Histogram of the Cur_aq_ (8 µg/mL)-treated *Klebsiella pneumoniae* cells labeled with DiSC_3_-5. The panel demonstrates the considerable right-shifting of the cell population (74.4%) that took up DiSC_3_-5, indicating significant depolarisation. (**E**) Histogram of Cur_aq_-meropenem (8 µg/mL + 2 µg/mL)-treated *Klebsiella pneumoniae* cells labeled with DiSC_3_-5. The panel demonstrates the significant right-shifting of the cell population (91.4%) that took up DiSC_3_-5, indicating massive depolarisation. (**F**) Histogram of Cur_aq_-meropenem (8 µg/mL + 4 µg/mL)-treated *Klebsiella pneumoniae* cells labeled with DiSC_3_-5. The panel demonstrates the cell population’s significant right-shifting, encompassing 96.0% of the total cell population that took up DiSC_3_-5, indicating colossal depolarisation.

The results of efflux inhibition and perturbed electric potential were very encouraging; therefore, we evaluated Cur_aq_ and meropenem’s capacity for fostering membrane depolarisation (i.e., triggering the decrease in membrane potential). To explore if Cur_aq_ combined with meropenem resulted in membrane depolarisation, flow cytometry-based investigations were executed, employing the membrane potential-sensitive dye, DiSC_3_-5. The dye DiSC_3_-5 partitions in the polarised cells showed reduced/quenched fluorescence. However, it failed to partition in the depolarised membrane; hence, fluorescence intensity increased. Therefore, the untreated control cells produced a low signal intensity, whereas the depolarised and leaky cells produced a high signal intensity. The meropenem and Cur_aq_ alone fostered membrane depolarisation in 33.2% and 74.4% of the total bacterial cell population, respectively (Figure 12C,D). Strikingly, exposure to the combination enabled a colossal increase in the depolarised/leaked cell population (0.6% to 96.0%) compared to the untreated cells (Figure 12A,E,F). The meropenem and Cur_aq_ combination results were comparable to the CCCP-treated cells (Figure 12B). For area selection, see Supplementary Information (Supplementary Information S8). 

## 3. Discussion

*K. pneumoniae* is one of the primary agents responsible for nosocomial infections across the globe. It harbors a wide array of virulence and pathogenicity factors, e.g., the capsule, lipopolysaccharide, carbapenemase, efflux systems, adhesins, etc. [18]. Globally, the rapid surge in the incidence of *Klebsiella* infections in recent years indicates the indiscriminate use of antibiotics that has fostered the emergence of multidrug resistance among *K. pneumoniae*. With time, *K. pneumoniae* isolates have exhibited augmented carbapenemases and efflux pump expression, indicating the most common reasons for its multidrug resistance [6,8,19,20,21]. 

In *K. pneumoniae,* mainly AcrAB and mdtK efflux systems are operative, belonging to the resistance-nodulation-division (RND) and multi-antimicrobial extrusion (MATE) families of efflux pumps, respectively [22]. The AcrAB-TolC efflux system is responsible for imparting resistance against quinolones, tetracyclines, and chloramphenicol in various MDR isolates. Some reports suggest their role in conferring resistance against cephalosporins and carbapenems [10,23,24,25]. Currently, carbapenems are the drugs of last resort used to treat and manage Gram-negative isolates. Among all the *Enterobacteriaceae* members, the most MDR has. been observed in *Klebsiella pneumoniae* isolates [8,26,27]. Therefore, molecular typing and analyses of these clinical isolates’ major virulence determinants may lead to more in-depth insights into multidrug-resistant *Klebsiella pneumoniae* infections. Enterobacterial repetitive intergenic consensus (ERIC) PCR was utilised to assess clonal relatedness in the present study. ERIC sequences occurred in extragenic regions of bacterial genomes in multiple copies in *Enterobacteriaceae* members and are imperfect palindromes of 127-bp. ERIC-PCR has widely been used as a method for distinguishing bacterial isolates. It is believed that the positions of copies of the ERIC sequence vary among different isolates of the same bacteria [28]. Therefore, the variation can easily be documented using amplification products when running on an agarose gel. Out of 97 multidrug resistant *K. pneumoniae* isolates in the current study, ERIC-PCR revealed 79 unique patterns. The present study’s observations agreed with Lai et al., who reported high heterogeneity due to variations in nucleotide sequences among *K. pneumoniae* isolates [29]. However, in the present study, ERIC-PCR genotypic analyses failed to reveal meaningful correlations with resistance patterns of *K. pneumonia.* Therefore, the current study also highlights the inadequacy of ERIC genotyping in predicting resistance patterns of *K. pneumoniae.* However, ERIC-PCR was sufficient for highlighting clonal similarities or differences, as in this method, two or more band differences are used as cut-offs for distinction. 

Furthermore, the resistance patterns and major virulence determinants among these isolates were investigated. In the present study, 82.38% of the total isolates of *K. pneumoniae* exhibited the multidrug resistance phenotype. However, in other studies carried out, the prevalence of MDR *K. pneumoniae* isolates was found to be 71.1% [19]. The astoundingly high rates of antimicrobial resistance detected in our study can be attributed to unrestricted use of antibiotics. 

In the present study, a total of 74 isolates (76.29%) were resistant to both imipenem and meropenem. In this context, it is essential to highlight the significant increase in carbapenem resistance among *K. pneumoniae* isolates in India and the western world over time. As highlighted by Hanna Sidjabat et al., as much as 70% of *Klebsiella pneumoniae* isolates were found to be KPC-producing [30]. Similarly, as per the Centre for Disease Dynamics, Economics and Policy (CDDEP) reports, up to 80% of all K. pneumoniae isolates are resistant to cephalosporins. Up to 60% of isolates are nonsusceptible to carbapenems in India. As per the data highlighted by Veeraraghavan et al., the percentage of carbapenem resistance among *Enterobacteriaceae* members in India in 2010 was 44%, whereas it was only 9% in 2008 [31]. This rapid upsurge in carbapenem resistance is indeed a serious concern not for India but also for the whole world. Specifically, *K. pneumoniae* has evolved a wide array of mechanisms (e.g., AmpC production, ESBL production, carbapenemase production, etc.) [32]. In the present study, a total of 65 (41.40%) isolates (of 157) were found to be metallo beta-lactamase producers, while 31 (19.51%) isolates were found to be producers of serine carbapenemases. Similarly, 61 (38.85%) isolates were found to be non-carbapenem producers. However, many of these isolates were also resistant to meropenem and imipenem (which are carbapenems), indicating other resistance modes.

Treatment options against these carbapenemase-producing *K. pneumoniae* isolates are minimal, and can include colistin or minocycline [33]. India’s minocycline resistance trend is comparable with meropenem; therefore, minocycline can be incorporated into the treatment regime as a carbapenem-sparing agent [34]. Similarly, owing to toxicity, the use of colistin is limited. However, due to a recent scarcity of treatment options, the use of colistin has been permitted for acute and life-threatening infections caused by *Pseudomonas aeruginosa* and *Acinetobacter baumannii* [34]. Interestingly, in the present study, all the tested *K. pneumoniae* isolates were sensitive to colistin (as investigated through the micro broth dilution method). 

Similarly, for adherence, type 1 and type 3 fimbrial adhesins were the primary requirements among *Enterobacteriaceae* [35]. In the current study, the FimH-1 gene was present in all pus and blood isolates. However, FimH-1 was found to be less prevalent among urine isolates. Similarly, irrespective of the source of isolation, the RmpA gene was present among all the tested isolates, indicating the inevitable role of capsular polysaccharides in pathogenicity [36].

In all, the present study results suggest a need for an increased search for molecules or tailored/fabricated compounds that may be effective against major virulence factors like the production of carbapenemases, efflux systems, and biofilm formation. Furthermore, additives must be sought for their synergistic relationships with and ability to improve the efficacy of existing drugs.

Over time, the course of the development of antibiotics has significantly changed. For instance, more than 20 different antibiotics were synthesised/extracted/formulated and then approved between 1940 and 1962 (22 years, an average of 1 antibiotic per year). However, between 1962 and 2010, only two new antibiotics made it to the market. Therefore, newer antibiotics’ paucity and an unprecedented increase in drug-resistant strains compel us to look for other alternatives, e.g., phytochemicals or repurposing existing antibiotics [37,38].

The present study sought to understand the contribution of efflux pumps in the current prevalence of multidrug resistant *K. pneumoniae* from our tertiary care health center. Further, the study aimed to evaluate the effects of Cur_aq_ over the efflux status and mechanistic underpinnings of Cur_aq_-mediated efflux inhibition of the tested pathogens. The incidence of carbapenem resistance, especially against meropenem, has increased. To ensure efflux pumps’ role among these resistant isolates, three different efflux pump inhibitors were utilised: CCCP, verapamil, and valinomycin. All these EPIs have reduced the MIC of meropenem. However, the MIC of meropenem among 13, 20, and 3 of the tested isolates remained unperturbed even after adding CCCP, verapamil, and valinomycin, respectively.

Interestingly, in the present study, after adding Cur_aq_, all the tested *Klebsiella* isolates showed a reduction in the MIC of meropenem. Earlier, only 9 (9.27%) *Klebsiella* isolates were found to be sensitive to meropenem; however, after adding Cur_aq_ (8 µg/mL), 85 (87.62%) isolates became sensitive to meropenem. The reduction in MIC to the susceptibility breakpoint implies the efflux pumps’ involvement in resistance to antibiotics. Interestingly, among carbapenems, ertapenem was more effective than imipenem and meropenem. However, the increase in susceptibility of meropenem post-supplementation endorsed the involvement of efflux pumps; as soon as Cur_aq_ blocked the efflux activity, the inhibitory activity of meropenem grew more pronounced. 

Subsequently, we quantified the degree of antimicrobial synergy for Cur_aq_ (Bliss coefficient) against *Klebsiella pneumoniae* at different concentrations, combined with increasing meropenem concentrations. We considered positive values the marker of the synergistic combination; negative values demonstrated antagonistic effects. The synthesised Cur_aq_ exhibited complete synergy over the tested range of meropenem concentrations. Interestingly, the highest synergy of Cur_aq_ was noted at 8 µg/mL concentration. Interestingly, while the Bliss independence model indicated that some measurable synergy levels were observed at all tested Cur_aq_ tested range, the FIC indices indicated synergy for the isolates against which the MIC of Cur_aq_ was found to be 8 µg/mL. 

However, the reduction in MIC in combination supports synergy. This may be important for medical use and dose optimisation. It is well-documented that meropenem imparts cellular toxicity, and if its effective dose gets reduced, it may alleviate meropenem-mediated cytotoxicity [39]. Furthermore, the lower concentration and aqueous solubility could have tremendous utility in the complex microenvironments of the lungs. The lower dose of additives and antibiotics could also minimise the risks of developing further antibiotic resistance.

The most prevalent efflux systems in *K. pneumoniae* belong to resistance-nodulation-division (RND) and multi-antimicrobial extrusion (MATE) families [22]. The MIC reduction—post-supplementation of Cur_aq_ and other EPIs—confirmed the efflux system’s role. Therefore, to validate our findings, we further typed the *Klebsiella* isolates for the most common efflux reported for carbapenem resistance, the AcrAB-TolC system. The AcrAB-TolC system is a tripartite assembly comprised of three distinct parts spanning across different layers: a periplasmic component (AcrA), a secondary transporter located in the inner membrane (AcrB), and the outer membrane channel (TolC) [40]. Recently, Padilla et al. and Wasfi et al. reported functional synergy between efflux pumps and various virulence determinants among *Klebsiella pneumoniae* [10,41]. Reports suggest that for upregulation of many genes involved in invasion and survival of *Klebsiella pneumoniae*, the AcrAB-TolC efflux system’s activity is a prerequisite [11]. Therefore, studying the presence and prevalence of the AcrAB-TolC efflux system among *Klebsiella* isolates is prudent. In the present study, 87.62% of all the tested ICU isolates of *Klebsiella pneumoniae* were found to harbor the AcrAB-TolC efflux system. However, 12 strains had an incomplete AcrAB efflux pump system (i.e., either TolC or AcrAB was absent). Such a high prevalence of the AcrAB-TolC efflux pump system indicates a dire need to develop biocompatible efflux pump inhibitors to potentiate frontline carbapenem drugs—especially meropenem.

In the case of meropenem, we did not know its influx rate. We had to determine the efficiency of Cur_aq_ to inhibit meropenem efflux by using a different system in which efflux could be triggered conveniently (in bacterial cells preloaded with meropenem). Then, the efflux could be measured directly as a function of time. Similar approaches were recently published in a highly optimised format wherein the direct measurement of AcrAB-TolC mediated the Nile red, and 1,2′-dinaphthylamine efflux were measured [42]. For the current study, we utilised 1,2′-dinaphthylamine assays. As suggested by Bohnert et al., 1,2-DNA could be used to probe membrane and membrane transport due to its second emission maxima in the near-infrared region at 810 nm. At this wavelength, cellular autofluorescence is almost undetectable, and also, interference with substrates or competitors is hardly encountered [43]. The Cur_aq_-mediated efflux inhibition started at 1 µg/mL concentration, whereas more than 90% efflux was abolished at 8 µg/mL. This indicated the possibility of using Cur_aq_ as an anti-efflux agent.

After Cur_aq_-mediated efflux inhibition was confirmed, we checked the AcrAB-TolC efflux system’s expression profile in various permutations and combinations. We found that meropenem significantly trigger acrA, acrB, and tolC expression at all tested concentrations. However, comparatively, acrB expression was less pronounced after infusion with meropenem. Interestingly, Cur_aq_ alone inhibited the expression of all the tripartite assemblies of the AcrAB-TolC efflux system. However, even in the presence of meropenem—when the expression of acrA, acrB, and tolC was much exaggerated—Cur_aq_ downregulated their expression significantly, showing its promise as a better efflux pump inhibitor.

We further tested our hypothesis that Cur_aq_ would perturb bacterial energy homeostasis, leading eventually to efflux inhibition. The effect of Cur_aq_ on the bacterial membrane potential was investigated using flow cytometry and confocal laser scanning microscopy employing DiOC_2_(3)-based assay. We utilised the known protonophore CCCP as a positive control for depolarisation. The membrane potential collapsed over 96%. These results showed that our synthesised Cur_aq_ depolarised *Klebsiella pneumoniae* membranes by interfering with the proton gradient. Next, to evaluate if the perturbations in electric potential had depolarised the cells, we used DiSC_3_-5-based flow cytometry investigation. Results endorsed the previously discussed findings of altered membrane potential. Interestingly, the combination of Cur_aq_ and meropenem (8 µg/mL + 4 µg/mL) fostered colossal depolarization in *Klebsiella pneumoniae* (96.0%).

In all, the present study reports efflux-mediated resistance against meropenem among *Klebsiella pneumoniae* isolates. Additionally, the current research unveils the controlling strategy of the efflux—utilising our synthesised water-soluble curcumin.

## 4. Materials and Methods

### 4.1. Ethical Approval

The study was commended by the Institutional Ethical Committee of the Institute of Medical Sciences, Banaras Hindu University (Dean/2018/EC/594).

### 4.2. Bacterial Isolation and Culture Conditions

In the present study, 157 nonrepetitive, consecutive *K. pneumoniae* isolates from intensive care unit patients were collected from the routine bacteriology laboratory of the Department of Microbiology, Institute of Medical Sciences, Banaras Hindu University Varanasi, India, from March 2019 to December 2019. Definitive identification of collected isolates was performed using established biochemical tests as described earlier [44]. After identification, the *K. pneumoniae* isolates were stocked in brain heart infusion broth (BHI) (HiMedia, India) with 10% glycerol (Merck, Germany) at –80 °C. All isolates were freshly subcultured on cysteine lactose electrolyte deficient (CLED) agar (HiMedia, India) before every analysis. The *K. pneumoniae* isolates with nonsusceptibility to any carbapenem drugs (e.g., meropenem, ertapenem or imipenem) as outlined by the Clinical and Laboratory Standards Institute (CLSI, 2019) were defined as carbapenem-resistant *K. pneumoniae* (CRKP).

### 4.3. Antimicrobial Susceptibility Testing

Antibiotic susceptibility testing of *Klebsiella pneumoniae* isolates was performed according to the modified Kirby–Bauer disc diffusion method, utilising 17 antibiotics and inhibition zone diameters measured in millimeters and interpreted following C.L.S.I. guidelines (2019, described previously except for colistin) [17]. The following antibiotic discs (HiMedia, India) were used: (1) Cephalosporins: ceftazidime (caz, 30 µg), cefuroxime (cxm, 30 μg), and cefepime (cfm, 30 µg), (2) Carbapenems: ertapenem (erta, 10 μg), meropenem (Mero, 10 µg) and imipenem (Imi, 10µg), (3) Aminoglycosides: gentamicin (Genta, 10 µg), and amikacin (Amika, 30 µg), (4) Quinolones: ciprofloxacin (Cipro, 5 µg) and levofloxacin (Levo, 5 µg), (5) Penicillins: Ampicillin (amp, 10 μg), Amoxicillin/Clavulanic acid (amc, 20/10 μg), Piperacillin-tazobactam (ptz, 110/10 μg) (6) Monobactams: aztreonam (azm, 30 µg), (7) Tetracycline (tetra, 30 μg), (8) Trimethoprim-Sulfamethoxazole (cot, 23.75 + 1.25 μg), and (9) Colistin (10 μg). In the present study, we defined multidrug resistance among isolates (MDR) if an isolate was found to be nonsusceptible to at least one agent in three or more antimicrobial classes.

### 4.4. Detection of Carbapenemases

The mCIM and eCIM phenotypic assays were performed using the approach described elsewhere with minor modifications [45,46]. Briefly, 1 µL loopful of the freshly cultured test *Klebsiella pneumoniae* isolates with suspected carbapenemase activity was resuspended in two microcentrifuge tubes containing 2 mL of brain heart infusion broth (BHI), and the bacterial suspension was gently vortexed for 15 s. For mCIM execution, one microcentrifuge tube was devoid of EDTA, while the other was supplemented with 10 µL EDTA (5 mM) (eCIM). A meropenem disk (10 µg) was gently added to each tube, and the tubes were incubated statically at 35 °C in ambient air for 4 h. After 4 h, the disks were taken out of the tubes and placed on cation-adjusted Mueller–Hinton agar plates and freshly lawn cultured (just before completion of the stipulated incubation) with carbapenem-susceptible reporter *E. coli* ATCC 25922 strain. The plates were incubated at 35 °C in ambient air for 24 h before the zone sizes were documented. In cases of negative mCIM results, the eCIM result was not read and reported. Zone size ≥ 19 mm was interpreted as negative mCIM, while zone diameters between 5–16 mm were considered mCIM positive in this study.

Zone sizes between 16–18 mm were interpreted as intermediate. The presence of pinpoint colonies in the zone of growth inhibition in the intermediate range (16–18 mm) was also considered a positive mCIM result. Similarly, eCIM was deemed positive when the zone diameter was ≥5 mm increased compared to the positive mCIM zone size.

### 4.5. Molecular Typing of Klebsiella Pneumoniae Isolates Using Enterobacterial Repetitive Intergenic Consensus (ERIC) PCR

The genetic profiles and clonal connotations among all carbapenem-resistant clinical *K. pneumoniae* isolates were appraised by polymorphism analysis of genomic DNA employing two primers, ERIC-1 5′-TGTAAGCTCCTGGGGATTAAC-3′ and ERIC-2 5′-AAGTAAGTGACTGGGGTGAGCG-3′ for repetitive intergenic enterobacterial consensus PCR typing, as described previously with minor modifications [47]. The typing was performed in cycles with an initial denaturation at 94 °C for 15 min followed by 40 cycles. Each cycle consisted of 1 min at 94 °C for denaturation, 1 min for primer annealing, and an extension at 72 °C for 8 min. ERIC fragments were visualised by 1.5% *w*/*v* agarose gel electrophoresis. Two observers visually analysed the amplification profiles, and their observations were transformed into binary data in a matrix, according to the presence (1) or absence (0) of bands. To evaluate the isolates’ genetic relationships, the matrix was subjected to cluster analysis by the nearest neighbor method (UPGMA) based on the Jaccard coefficient for automatic generation of the dendrogram using the software NTSYSpc version 2.0 (NY, USA). A previously identified nosocomial strain of K. pneumoniae encoding the bla-KPC gene was used as a positive control. As a negative control, *Staphylococcus aureus* strain ATCC 43300 was used.

### 4.6. Synthesis of Water-Soluble Curcumin

#### 4.6.1. Synthesis of Galactose Azide (2,3,4,6-Tetra-O-Acetyl-β-D-Galactopyranosyl Azide) (**2**)

Acetylated galactose (4.0 g, 10.25 mmol) was poured in dry DCM, maintaining the temperature at 0 °C. Hydrogen bromide solution (HBr 33% *w*/*w* in acetic acid) 12.0 mL was added slowly under anhydrous conditions, and the reaction mixture was stirred for 3–4 h at 0˚C to 10 °C. After completing the reaction (monitored by TLC), the reaction mixture was extracted with DCM and neutralised by NaHCO_3_. The organic layer was collected, dried over sodium sulphate and evaporated under reduced pressure to obtain per-*O*-acetylated galactose bromides. Next, the obtained anomeric bromide (3.80 g, 9.24 mmol) was dissolved in dry DMF followed by the addition of sodium azide (1.8 g, 3.0 equivalent). Then, the reaction was stirred at 80 °C for 5 h. After completion of the reaction monitored by TLC, the solvent was evaporated, and the resulted residue was extracted with EtOAc. After washing with ice water, subsequent evaporation of the organic layer occurred. The generated crude mass was loaded over column chromatography to obtain the pure product (2.55 g, 74%) (Figure 2a, Supplementary Figure S1).

White solid, 2.55 g, yield 74%; *R_f_* = 0.4 (40% ethyl acetate/*n*-hexane); ^1^H NMR (500 MHz, CDCl_3_): δ 5.41 (d, *J* = 4.0 Hz, 1H), 5.17–5.13 (m, 1H), 5.04–5.01 (m, 1H), 4.59 (d, *J* = 7.0 Hz, 1H), 4.19–4.12 (m, 2H), 4.02–3.99 (m, 1H), 2.16 (s, 3H), 2.08 (s, 3H), 2.05 (s, 3H), 1.98 (s, 3H); ^13^C NMR (125 MHz, CDCl_3_): δ 170.3, 170.0, 169.9, 169.3, 88.2, 72.8, 70.7, 68.0, 66.8, 61.2, 20.6, 20.5, 20.4 ppm.

#### 4.6.2. Synthesis of Curcumin Di-Alkyne (**4**)

A round bottom flask was charged with the mixture of curcumin (1.0 g, 2.71 mmol) and K_2_CO_3_ (0.75 g, 5.42 mmol, 2.0 equiv.) in dry DMF under argon atmosphere, followed by addition of propargyl bromide (0.54 mL, 6.78 mmol) and then stirred at room temperature for 48 h (Figure 2a). After the completion of the reaction (monitored by TLC), the reaction mixture was concentrated under reduced pressure to obtain crude mass. Next, the crude mass was subjected to column chromatography (ethyl acetate: hexane) to obtain the curcumin-di-alkyne. Yield (55%); Orange-red-colored semisolid, R*_f_* = 0.5, (15% ethyl acetate/*n*-hexane); ^1^H NMR (500 MHz, CDCl_3_): δ 7.70–7.53 (m, 2H), 7.15–6.93 (m, 6H), 6.72–6.45 (m, 2H), 4.74 (d, *J* = 6.5 Hz, 4H), 3.88–3.84 (m, 6H), 3.15–3.11 (m, 2H), 2.51–2.49 (m, 2H); ^13^C NMR (125 MHz, CDCl_3_): δ193.3, 193.2, 183.0, 182.4, 149.6, 149.4, 145.4, 145.1, 144.8, 141.9, 140.1, 130.9, 130.5, 129.3, 129.0, 127.9, 127.8, 123.4, 123.1, 122.3, 121.9, 121.6, 118.5, 118.3, 115.9, 115.1, 113.66, 113.60, 133.3, 110.8, 110.7, 110.5, 101.3, 78.8, 77.9, 77.6, 76.2, 76.1, 72.0, 70.5, 66.8, 63.0, 56.46, 56.44, 55.9, 55.8 and 55.7 ppm. I.R. (cm^−1^) 3511.29, 3378.49, 3015.33, 2845.98, 1843.84, 1627.15, 1602.92, 1504.47, 1278.88.

#### 4.6.3. Synthesis of Curcumin Clicked Di-Acetylated Galactose (**5**)

Curcumin di-alkyne(0.5 g, 1.12 mmol) was dissolved in 20 mL of THF/water (1:1), followed by addition of 2,3,4,6-Tetra-O-acetyl-*β*-d-galactopyranosyl azide (1.25 g, 3.37 mmol, 3.0 equiv.) in presence of CuSO_4_·5H_2_O (224 mg, 0.899 mmol, 0.8 equiv.) and sodium ascorbate (178 mg, 0.899 mmol, 0.8 equiv.) (Figure 2a, Supplementary Figure S2). The mixture was stirred at room temperature for 12 h. Reaction monitoring was executed by TLC; after the completion of reaction on TLC, reaction mixture was concentrated under reduced pressure to obtain crude. Purification of crude mass by flash chromatography (ethyl acetate: hexane) afforded 1,4-disubstituted triazole derivative, yield 63%, isolated through column chromatography. ^1^H NMR (500 MHz, CDCl_3_): δ 7.93 (s, 2H), 7.72–7.42 (m, 4H), 7.14–6.99 (m, 4H), 5.83 (d, *J* = 8.5 Hz, 2H), 5.52–5.51 (m, 4H), 5.28–5.22 (m, 6H), 4.22–4.11 (m, 10H), 3.90–3.86 (m, 6H), 2.19 (s, 6H), 2.01–1.96 (m, 12H), 1.82–1.81 (m, 6H); ^13^C NMR (125 MHz, CDCl_3_): δ 193.9, 183.1, 170.2, 170.1, 169.8, 169.7, 168.9, 150.2, 149.6, 149.0, 144.2, 144.1, 142.1, 139.1, 132.0, 131.9, 129.0, 128.5, 128.4, 123.8, 122.5, 121.6, 120.5, 119.0, 118.4, 133.9, 113.8, 113.6, 110.9, 110.5, 86.1, 73.9, 70.6, 67.7, 67.6, 66.8, 62.78, 62.71, 61.1, 61.0, 56.0, 56.9, 20.5, 20.3, 20.1, and 20.0 ppm. I.R.(cm^−1^) 3422.7, 2924.86, 2854.44, 1742.2, 1639.26, 1465.26, 1095.12.

#### 4.6.4. Synthesis of De-O-Acetylated Curcumin Di-Galactose (**6**)

Per-*O*-acetylated-galactose-linked curcumin derivative (200 mg) was dissolved in a mixture of dry MeOH/dry THF/dry DCM (8 mL in the ratio 3:0.5:0.5 respectively). After that, the solution of NaOMe (1 M)—typically 40–50 µL—was added, and the reaction mixture was stirred for 48 h (Figure 2a, Supplementary Figure S2). After the reaction was complete, the solution was completely evaporated, then diluted with distilled water. This was followed by neutralization (6–7 pH) using ion exchange resin (Amberlite IR 120 H^+^). Resin was filtered out and the resulting solution was condensed to achieve compound (6) with 88% yield. ^1^H NMR (500 MHz, CDCl_3_): δ 8.12 (s, 2H), 7.27–6.75 (m, 4H), 6.16 (d, *J* = 16.0 Hz, 1H), 5.50 -5.48 (m, 2H), 5.02–4.96 (m, 2H), 4.04 (br, 2H), 3.90 (br, 3H), 3.79–3.37 (m, 22H); ^13^C NMR (125 MHz, CDCl_3_): δ 182.2, 181.5, 175.7, 171.1, 164.0, 148.7, 147.6, 143.3, 140.5, 129.4, 126.5, 124.4, 123.4, 122.7, 121.7, 116.1, 113.9, 110.5, 88.1, 78.3, 72.9, 72.1, 69.7, 68.5, 62.5, 61.6, 61.5, 60.8 and 55.6 ppm. I.R. (cm^−1^); 3226.15, 2924.59, 2853.98, 1634.99, 1567.74, 1411.43, 1096.70.

### 4.7. Photophysical Behavior and Solubility

The absorbance profile was evaluated using a UV-Vis absorption spectrometer (PerkinElmer, Waltham, Massachusetts, USA) with a 10 mm optical path length. The aqueous solubility of the synthesised curcumin derivatives (compared to the native form) were assessed by UV-Vis spectrophotometry. We first went for general spectral scanning and then prepared the calibration curve by plotting the concentrations versus absorbance at λmax. Then, the slope was calculated from the plot. We then dissolved the maximum dissolvable amount into MilliQ water, and the remaining undissolved part was removed by centrifugation. At the same λ_max_, we determined the absorbance of unperturbed MilliQ. We then calculated the solubility by applying the following Equation (3):

(3) $$Solubility=\left(Absorbanceat\lambda max\right)/Slope$$

### 4.8. Minimum Inhibitory Concentration (MIC) Determination

#### 4.8.1. Antibiotics and Efflux Inhibitors

We employed ten antibiotics, namely meropenem, meropenem/sulbactam, imipenem, ertapenem, amikacin, gentamicin, levofloxacin, ciprofloxacin, ceftazidime, and ceftazidime/avibactam. Additionally, we also used carbonyl cyanide m-chlorophenylhydrazine (CCCP) as a protonophore along with verapamil and valinomycin as efflux pump inhibitor (EPI) and proton pump inhibitor (PPI), respectively. Water-soluble curcumin (Cur_aq_) was also utilised. Stock solutions of Cur_aq_, verapamil, valinomycin were prepared in MilliQ water while CCCP was dissolved in dimethyl sulfoxide (DMSO). However, we used fixed sub-inhibitory concentrations of soluble curcumin, CCCP, verapamil, and valinomycin (8, 10, 128, and 1 µg/mL, respectively) to delineate their possible effects on the resistance profile of meropenem.

#### 4.8.2. Phenotypic Evaluation of Drug Synergy

We compared the synergism phenotypically, first by diluting the agar with a fixed sub-inhibitory concentration of curcumin (128 µg/mL) and the water-soluble variant of curcumin (8 µg/mL), with simultaneous evaluation of changes in the zone of inhibition of the in-vogue drugs (employing modified Kirby–Bauer method).

#### 4.8.3. Quantifying Synergy Using the Bliss Independence Model and Calculating the Fractional Inhibitory Concentration Index

Drug synergism was further calculated using the Bliss Independence Model described previously, using the below-mentioned Equation (4) [17]:

(4) $$S=\left(Fxo/Foo\right)\left(Fyo/Foo\right)-\left(Fxy/Foo\right)$$

*Fxy* refers to the growth rate in the combination of test compounds at the concentration *X*, Cur_aq_, and *Y* for the meropenem. Similarly, *Fxo* and *Fyo* refer to the growth rates in the presence of the Cur_aq_ and meropenem at concentrations *X* and *Y*, respectively. Foo refers to the growth rate in the absence of Cur_aq_ and meropenem. *S* is the degree of synergy that establishes a synergistic interaction for its positive values and an antagonistic interaction for its negative values. At various time points, growth rates were ascertained by calculating the slope of the growth curve being analysed for 4.5 h, as described previously [44].

The FIC index was calculated as described earlier [48]. Briefly, the FIC index was calculated for the first well with no perceivable growth (i.e., non-turbid well) for each row and column, representing the Cur_aq_-meropenem combinations that inhibited the *K. pneumoniae* growth. We considered OD_600_ values of 0.01–0.02 as the marker of a non-turbid well in case of any confusion. The following Equation (5) determined the FIC:

(5) $$FIC=\frac{CombinedMICofCuraq\wedge meropenem}{MICofsCuralone}+\frac{CombinedMICofCuraq\wedge meropenem}{MICofmeropenemalone}$$

Subsequently, we determined the FIC index median and its range. The values were interpreted as per standard protocol. The FIC index value of ≤0.5 indicated synergism, while the values ranging >0.5 to 1 suggested additive behavior. Similarly, values ranging from 1–4 were interpreted as indifferent, while values >4 depicted antagonism.

### 4.9. Determination of Various Virulence Factors

The virulence factors investigated were related to the carbapenemase production (bla__KPC_), biofilm formation and adherence (RmpA, FimH-1). For PCR-based detection, the following primers were used (Table 6).

### 4.10. Detection of Major Efflux Pumps System

The AcrAB-TolC efflux system’s prevalence among all the meropenem-resistant clinical *K. pneumoniae* isolates was established by amplicon analysis (Table 6) as outlined previously with minor modifications [41].

### 4.11. Mechanistic Underpinnings

#### 4.11.1. 1,2′-Dinaphthylamine (1,2′-DNA) Efflux Assay

1,2′-dinaphthylamine (1,2′-DNA) efflux assay was performed as described previously with minor modifications [43]. Briefly, the exponential phase cells (1 mL) were harvested and resuspended in 1 mL phosphate-buffered saline (PBS, pH 7.2). Subsequently, the cells were pelleted, rinsed, and resuspended in PBS, followed by exposure with 8 µg/mL CCCP with shaking at 240 rpm for 20 min at 35 °C. Then, 1,2′-DNA (20 µg/mL) was added and incubated for 1 hr at 37 °C, secluded from light to ensure its minimal degradation and maximum uptake. The exposed cells were then centrifuged at 13,000 *g* for 2 min at 25 °C to remove excess CCCP and 1,2′-DNA. The pellet was resuspended in the same freshly prepared PBS volume (pH 7.2) containing 1 mM MgCl_2_. To the suspension obtained, 1, 2, 4, or 8 µg/mL of water-soluble curcumin (Cur_aq_) was added. However, to trigger efflux, glucose (0.1% *w*/*v*) was infused into the wells. The 1,2′-DNA efflux was determined by measuring its fluorescence for 60 s with λ_exi_/λ_emi_ 370/810 nm. The 1,2′-DNA fluorescence was normalised to 100 relative fluorescence signals of the untreated/preenergized cells and was utilised as the negative control.

#### 4.11.2. Quantitative Real-Time PCR (qRT-PCR) for Determination of Expression of acrA, acrB and TolC in Presence and Absence of Soluble Curcumin or CCCP, Along with Meropenem at Different Concentrations

Total RNA was isolated by the Trizol-based method described previously [54]. After 45 min of treatment with Cur_aq_ (8 µg/mL) or CCCP (10 µg/mL) along with meropenem at different concentrations (0.5–4 μg/mL), total RNA was extracted using Trizol^®^ reagent (Ambion, USA). Total RNA was first digested with RNAse free DNase (Fermantas, Germany) to avoid DNA contamination before use. Isolated RNAs were immediately preserved at −80 °C. For cDNA preparation, 1 μg total RNA was subjected to reverse transcription employing the cDNA conversion kit (Qiagen, Germany). The expression of genes involved in the efflux (AcrAB-TolC) was assessed using SYBR green master mix (Thermo Scientific, Waltham, Massachusetts USA) on ABI 7500 Fast system (Applied Biosystems) using 5 pmol/μL of specific primers with *rpoB* as an endogenous control (Table 1). The abundance/decline of RNA was normalised to the geometric average of endogenous control *rpoB* for ΔCt. The fold change (ΔCt) was calculated as the difference between the expression of AcrAB-TolC in the presence and absence of Cur_aq_, CCCP, meropenem (0.5–4 μg/mL), and Cur_aq_ with meropenem (8 µg/mL + 4 µg/mL). We incorporated three biological replicates and two technical replicates for the tested genes, and their relative expression was subsequently calculated by the 2^−∆∆ct^ method.

#### 4.11.3. Spectrophotometric Analysis of Meropenem

The concentration-dependent spectral analysis of meropenem (50, 100, and 200 µg/mL) (TCI, Japan) was performed using an H1 synergy multimode reader at room temperature (approximately 25 °C) in a UV-transparent 96-well plate (Nunc, Denmark). Measurements with bandwidths of 5 nm were implemented for analysing absorption spectra. Subsequently, we plotted the concentration versus absorbance plot for meropenem at 295 nm.

#### 4.11.4. Meropenem Hydrolysis by Bacterial Cells

A hundred microliters of either meropenem solution (32 μg/mL) or PBS was mixed with 100 μL of each bacterial suspension (1.5 ×10^8^ CFU/mL) and incubated for 60 min at 37 °C. Subsequently, the absorbance was read in the range of 220–400 nm. *Klebsiella pneumoniae* ATCC 4352 served as the control for the non-carbapenemase producer, while ATCC BAA-1705 served as the control for carbapenemase production.

#### 4.11.5. Estimation of Background Absorbance

We selected ten representative isolates with high MIC against meropenem (≥33 µg/mL). A bacterial suspension of each strain was prepared at an optical density at 600 nm of 3.0. Two-fold serial dilutions (64× dilution) were performed to generate bacterial suspensions at eight different concentrations. The control comprised PBS without bacteria. A hundred microliters of the bacterial suspension were mixed with 100 μL of meropenem solution (32 μg/mL) or PBS. Absorbance was measured at 295 nm after incubation at 37 °C for 60 min. The experiment was done in triplicate.

#### 4.11.6. Quantification of Bacterial Meropenem Hydrolytic Activities

Five meropenem-resistant isolates within each MIC category (except 256 µg/mL) were tested for meropenem hydrolysis. Strains were cultured on CLED agar plates overnight, and 0.5 McFarland’s match bacterial suspension was made in 100 μL of meropenem solution (64 μg/mL) directly. The incubation was made at 37 °C for 30, 60, and 120 min and the absorbance of each reaction mixture was read at 295 nm.

The percent hydrolysis was calculated as follows Equation (6):

(6) $$\begin{array}{ll} \%\;hydrolysis= & 1-(Absorbance\;@\;295\;nm\;at\;the\;time\;of\;incubation\\ & -Absorbance\;@\;295\;nm\;after\;defined\;time\;of\;incubation)\\ & /\left(Absorbance\;of\;meropenem\;at\;that\;concentration-Absornabce\;of\;PBS\right)\;\times \;100 \end{array}$$

#### 4.11.7. Quantification of the Effect of Cur_aq_ on Bacterial Meropenem Hydrolytic Activities

Five meropenem resistant strains within each MIC category (except 256 µg/mL) were utilised for this study. Strains were cultured on CLED agar plates overnight, and 0.5 McFarland’s match bacterial suspension was made in 100 μL of meropenem solution (64 μg/mL) directly. The incubation was made at 37 °C for 30, 60, and 120 min in the presence of different concentrations of Cur_aq_ (4, 8, 16, 32 µg/mL) and the absorbance of each reaction mixture was read at 295 nm.

The percent hydrolysis was calculated as follows Equation (7):

(7) $$\begin{array}{ll} \%\;hydrolysis= & 1-(Absorbance\;@\;295\;nm\;at\;the\;time\;of\;incubation\\ & -Absorbance\;@\;295\;nm\;after\;defined\;period\;of\;incubation)\\ & /(Absorbance\;of\;meropenem\;at\;that\;concentration\;at\;295\;nm\;\\ & -Absorbance\;of\;Curaq\;at\;295\;nm-Absornabce\;of\;PBS)\;\times \;100 \end{array}$$

#### 4.11.8. Preparation of Cell-Free Extracts

Bacteria were harvested during the late logarithmic growth phase into 0.1 M Tris buffer (pH 8.0). Harvested cells were then centrifuged at 17,000× *g* for 8 min at 4 °C. The resulting pellet was washed thrice and resuspended in 0.1 M Tris buffer. Cells were lysed by repeatedly freezing and thawing the suspensions. The suspension was allowed to thaw completely before refreezing. To get crude extracts, the cell lysate was centrifuged at 27,000× *g* for 10 min at 4 °C, and the supernatant carefully separated and stored at −20 °C.

#### 4.11.9. Protein Measurement

Protein concentration was determined by the Bradford method. The bovine serum albumin was the standard used. The standard curve was used to determine the protein amount in the extracts (mg/mL).

#### 4.11.10. Inhibition Assays on Carbapenemases

Hydrolysis of meropenem by carbapenem was monitored using an H1 Synergy multimode reader by following the absorbance changes at 295 nm using Δε_295_ = −9023 M^−^^1^ cm^−^^1^. The reaction medium employed was 10 mM HEPES, pH 7.5, 200 mM NaCl, 50 μg/mL BSA, and 20 μM ZnSO_4_ at 30 °C. Reactions were carried out in tissue culture plates in a final volume of 200 μL, with the crude bacterial extract. Cur_aq_ was dissolved in MilliQ to a final concentration of 256 µg/mL and then serially diluted 2-fold (to 4 µg/mL). The assay was initiated by the addition of crude bacterial extract to the mixture of substrate and Cur_aq_.

#### 4.11.11. Measurement of the Electrical Potential

The electrical potential was measured via the membrane-permeable DiOC_2_(3) dye, as outlined previously with minor modifications [55]. Cells were first grown to the mid-log phase in BHI supplemented with either meropenem, Cur_aq_ or meropenem and Cur_aq_. A total of 1 mL mid-log phase growing treated *Klebsiella pneumoniae* cells was collected after centrifugation at 4500× *g* for 15 min and then resuspended in 1 mL of permeabilisation buffer [10 mM Tris–HCl (pH 7.6), 1 mM EDTA and 10 mM glucose]. The cells were incubated for 30 min with 2 μL DiOC_2_(3) dye (5 mg/mL) (Invitrogen) at room temperature. Subsequently, the cells were recollected at 4500× *g*. Then, the obtained cell pellet was resuspended in 500 μL of permeabilisation buffer. The stained bacterial suspension was then assayed in a flow cytometer (C6 BD Accuri, Becton–Dickinson, San Jose, CA, USA) equipped with laser excitation at 488 nm. Fluorescence was then monitored in the green and red channels. The forward scatter, side scatter, and fluorescence was collected with logarithmic signal amplification.

However, the findings of flow cytometry were further validated by confocal laser scanning microscopy. For this, the cells were dropped onto the glass slide and covered with the coverslip. The samples were then immediately examined using the Zeiss LSM 510 inverted confocal laser-scanning microscope (Carl Zeiss, Jena, Germany) fitted with a Plan-Neofluar 63X/1.3 oil objective with a z-step of 2.0 μm or 40X objective with a z-step of 5.0 μm and analysed using ZenBlue software (Carl Zeiss, Jena, Germany). The excitation was made at 488 nm, while the emission was recorded at 530 (green) and 610 (red) nm.

#### 4.11.12. Membrane Depolarisation Assay

The plasma membrane depolarisation of bacterial membranes was measured, employing potential-sensitive dye DiSC_3_-5 by steady-state fluorimetry and flow cytometry by the method described previously with minor modifications [16]. Briefly, the overnight-grown test *Klebsiella pneumoniae* isolates were diluted in BHI broth to the absorbance index of 0.05 at λ_max_ 600. This was followed by the addition of 50 µL of Cur_aq_ (8 µg/mL), meropenem (4 µg/mL), and their combinations for 2 h at 37 °C. Subsequently, 50 µL of DiSC_3_-5 (1 µM) was added and incubated for the next 30 min. Negative control was devoid of any drug/Cur_aq_ while a CCCP-treated bacterial panel acted as the positive control. The steady-state fluorescence was monitored and recorded at λ_exi/_λ_emi_~622 nm/670 nm (Synergy H1 Hybrid Multi-Mode Reader). However, membrane depolarisation was further validated by flow cytometry.

### 4.12. Statistical Analysis

The differences between samples (control and test isolates with and without the drug(s)) were computed with the help of one-way ANOVA followed by Dunnett’s and Tukey-Kramer (Tukey’s W) multiple comparison tests as and when required. However, in the experiment with protonophores, EPIs, and galactose-clicked curcumin, the fold change of ≥2 was considered significant. Differences were considered statistically significant at a 5% level when *p* < 0.05. GraphPad Prism version 8.2 was used for all statistical analysis and graph plotting.

## 5. Conclusions

In conclusion, Cur_aq_ seems to be a suitable candidate to extend studies further in the ongoing effort to find an altogether newer generation of RND efflux pump inhibitors that act by disrupting the proton motive force and membrane potential. Additionally, it shows its broad usefulness as an efflux pump inhibitor in combination with cell wall acting drugs like meropenem, due to its membrane permeabilising properties. Our results further revealed that the combination of Cur_aq_ and meropenem is highly synergistic against *Klebsiella pneumoniae*, reducing drug dose regimes and toxicity. Almost all efflux pump systems rely on energy (ATP); therefore, the drug that inhibits the PMF will inhibit ATP generation and eventually shut down the efflux system. Accordingly, being embodied with this property, Cur_aq_ can be utilised in newer putative adjuvants to in-use drugs, especially meropenem. However, further design of such molecules would be needed for better treatment outcomes in *K. pneumoniae* pathogenesis and to overcome the mechanisms of resistance.

## Figures and Tables

**Table 1:** mCIM and eCIM results for clinical isolates of *Klebsiella pneumoniae.*

Condition	Number of Isolates (*n* = 157)	Interpretation
mCIM **positive**, eCIM **positive**	65 (41.40%)	Member of Ambler Class **B**
mCIM **positive**, eCIM **negative**	31 (19.51%)	Member of Ambler Class **A or D**
mCIM **negative**, eCIM **negative**	61 (38.85%)	Non-carbapenemase producer

**Table 2:** The tally of the range of minimum inhibitory concentration (µg/mL) of meropenem, imipenem, and ertapenem— along with protonophore, efflux pump inhibitor, proton pump inhibitor, and soluble Curcumin (Cur_aq_)—against carbapenemase-producing clinical isolates of *K. pneumoniae* (*n* = 96).

Drug Concentration														
Drugs	<1 µg/mL	1 µg/mL	2 µg/mL	4 µg/mL	8 µg/mL	16 µg/mL	32 µg/mL	64 µg/mL	128 µg/mL	256 µg/mL	512 µg/mL	1024 µg/mL	>1024 µg/mL	
**Mero**	7	2	-	6	18	16	18	17	11	1	-	-	-	
**Imi**	5	6	-	13	20	19	14	8	9	2	-	-	-	
**Erta**	7	6	5	4	22	15	16	13	8	-	-	-	-	
**CCCP**	-	-	-	-	-	12	43	37	4	-	-	-	-	
**Vera**	-	-	-	-	-	-	-	-	-	42	31	23	-	
**Vali**	-	-	44	29	17	6	-	-	-	-	-	-	-	
**Cur_aq_**	-	-	-	-	-	14	82	-	-	-	-	-	-	

Mero, Imi, Erta, Vera, Vali, and Cur_aq_ stand for meropenem, imipenem, ertapenem, verapamil, valinomycin and water-soluble curcumin, respectively.

**Table 3:** The fold reduction in minimum inhibitory concentration (µg/mL) of meropenem combined with CCCP, verapamil, valinomycin, and soluble curcumin against clinical isolates of *K. pneumoniae* (*n* = 97) and their gross numbers.

Fold Reduction in MIC	Meropenem + CCCP (No of Isolates)	Meropenem + Verapamil (No of Isolates)	Meropenem + Valinomycin (No of Isolates)	Meropenem + Soluble Curcumin (No of Isolates)
**No Change**	13	20	3	-
**Δ 2**	3	36	21	3
**Δ 4**	8	27	42	24
**Δ 6**	19	11	23	51
**Δ 8**	26	2	6	13
**Δ 10**	17	-	1	5
**Δ 12**	8	-	-	-
**Δ 14**	2	-	-	-

**Table 4:** The change in the number of meropenem sensitive isolates when combined with CCCP, verapamil, valinomycin, and soluble curcumin against clinical isolates of *K. pneumoniae* (*n* = 97).

	Meropenem + CCCP (No of Isolates)	Meropenem + Verapamil (No of Isolates)	Meropenem + Valinomycin (No of Isolates)	Meropenem + Soluble Curcumin (No of Isolates)
**No of meropenem sensitive isolates without the addition**	9 (9.4%)	9 (9.4%)	9 (9.4%)	9 (9.4%)
**No of meropenem sensitive isolates after addition**	59 (60.82%)	36 (37.11%)	74 (76.28%)	85 (87.62%)

**Table 5:** Kinetic parameters of noncompetitive inhibition of carbapenemases by Cur_aq_ (I).

Parameters	Untreated	[I] = 4.46 µM	[I] = 9.34 µM	[I] = 18.69 µM	[I] = 37.38 µM
***K_m_* (µM)**	4.702	4.702	4.702	4.702	4.702
***V_max_* (µM/Sec)**	0.7742	0.727	0.628	0.659	0.647
***K_i_* (µM)**	-	8.741	9.063	23.02	45.75
**R^2^**	-	0.984	0.981	0.984	0.980
**% Inhibition**	-	33.7%	50%	44.86%	44.96%

**Table 6:** List of primers.

Gene	Sequence (5’→3’)	Amplicon Size (bp)	Melting Temperature (T_m_ in °C)	Reference
bla__KPC_	F: TGTCACTGTATCGCCGTC R: CTCAGTGCTCTACAGAAAACC	1010	60	[49]
*RmpA*	F: ACTGGGCTACCTCTGCTTCA R: CTTGCATGAGCCATCTTTCA	435	53	[50]
*FimH-1*	F: GCCAACGTCTACGTTAACCTG R: ATATTTCACGGTGCCTGAAAA	180	43	[41]
*AcrAB*	F: ATCAGCGGCCGGATTGGTAAA R: CGGGTTCGGGAAAATAGCGCG	312	53	[41]
*TolC*	F: ATCAGCAACCCCGATCTGCGT R: CCGGTGACTTGACGCAGTCCT	527	51	[41]
*rpoB*	F: AAGGCGAATCCAGCTTGTTCAGC R: GACGTTGCATGTTCGCACCCATCA	For Real-time		[51]
*Acr A*	F: GTCCTCAGGTCAGTGGCATTA R: ATTGCTCTGCTGCGCCGTT	For Real-time		[52]
*Acr B*	F: AAACTTCGCCACTACGTCATA R: AGCTTAACGCCTCGATCAT	For Real-time		[53]

## Data Availability

No new data were created or analysed in this study. Data sharing is not applicable to this article.

## Supplementary Materials

The following are available online at https://www.mdpi.com/article/10.3390/antibiotics10040388/s1 as additional data. Figure S1: ^1^H and ^13^C NMR spectrum of the synthesised compounds (**2**–**6**) (Supplementary information 1), Figure S2. IR spectra of the compounds **5** and **6** (Supplementary information 2), Figure S3: The mCIM and eCIM results for clinical isolates of *K. pneumoniae,* Figure S4: The minimum inhibitory concentration (µg/mL) of in-vogue antibiotics—along with protonophore, efflux pump inhibitor, proton pump inhibitor, and soluble curcumin—against clinical isolates of *K. pneumoniae,* Figure S5: Antibiogram, Figure S6: Dendrogram.
