# Supplementary material for: Galactose-Clicked Curcumin-Mediated Reversal of Meropenem Resistance among Klebsiella pneumoniae by Targeting Its Carbapenemases and the AcrAB-TolC Efflux System
Source: Antibiotics (Basel). 2021 Apr 4;10(4):388. doi: 10.3390/antibiotics10040388 (PMC8066637; doi:10.3390/antibiotics10040388)

Supporting Information

Galactose "clicked" Curcumin mediated reversal of meropenem resistance among *Klebsiella pneumoniae* by targetting its Carbapenemases and the AcrAB-TolC efflux system

Shivangi Yadav[1&], Ashish Kumar Singh[1, 4&], Anand K Agrahari[2], Akhilesh Kumar Pandey[3], Munesh Kumar Gupta[1], Dipshikha Chakravortty[4, 5], Pradyot Prakash[1$], Vinod Kumar Tiwari[2$],

[1]Bacterial Biofilm and Drug Resistance Research Laboratory, Department of Microbiology, Institute of Medical Sciences, Banaras Hindu University, Varanasi-221005, India

[2]Department of Chemistry, Institute of Science, Banaras Hindu University, Varanasi-221005, India

[3]Department of Biochemistry, Institute of Science, Banaras Hindu University, Varanasi-221005, India

[4]Department of Microbiology and Cell Biology, Indian Institute of Science, Bengaluru-560012, India

[5]Center for Biosystem Science and Engineering, Indian Institute of Science, Bengaluru-560012, India

[&] Equally contributed

[$] Corresponding author

Emails of corresponding authors: pradyot_micro@bhu.ac.in, Tiwari_chem@yahoo.co.in

Table of Contents:

**S1.** ^{1}H and ^{13}C NMR spectrum of the synthesized compounds (**2-6**) (Supplementary information 1)	3-11
**S2.** IR spectra of the compounds 5 and 6 (Supplementary information 2)	12-13
**S3.** The mCIM and eCIM results for clinical isolates of *K. pneumoniae*	14-20
**S4.** The minimum inhibitory concentration (µg/ml) of the in vogue antibiotics along with protonophore, efflux pump inhibitor, proton pump inhibitor, and soluble curcumin against clinical isolates of *K. pneumoniae*	21-27
**S5.** Antibiogram	28
**S6.** Dendrogram of isolated isolates after ERIC PCR	29

^{1}H and ^{13}C NMR Spectrum of synthesized compounds

^{1}H and ^{13}C NMR Spectrum of developed compound:

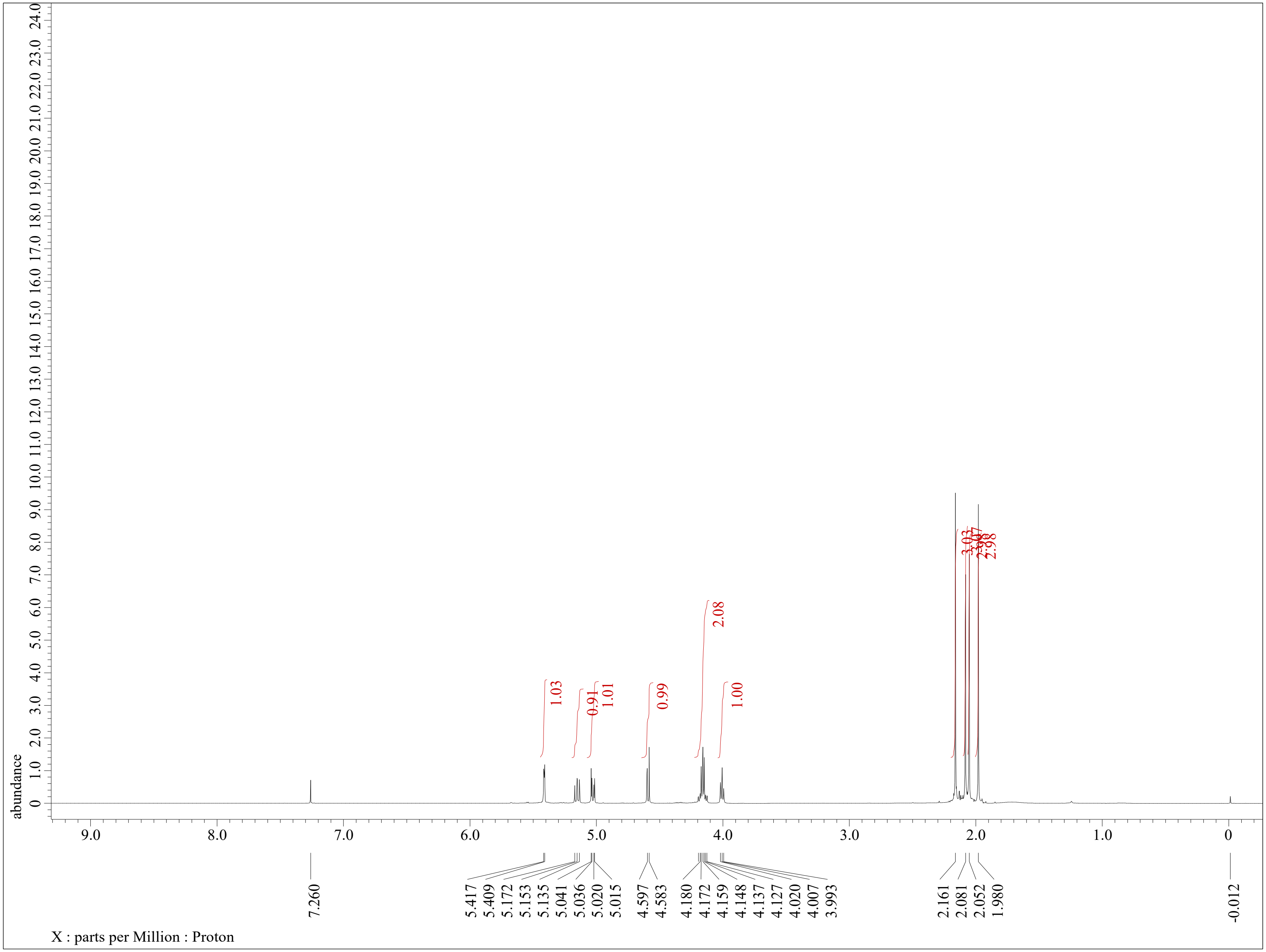

Spectra 1: ^{1}H NMR (500 MHz, $CDCl_3$) of compound **2**

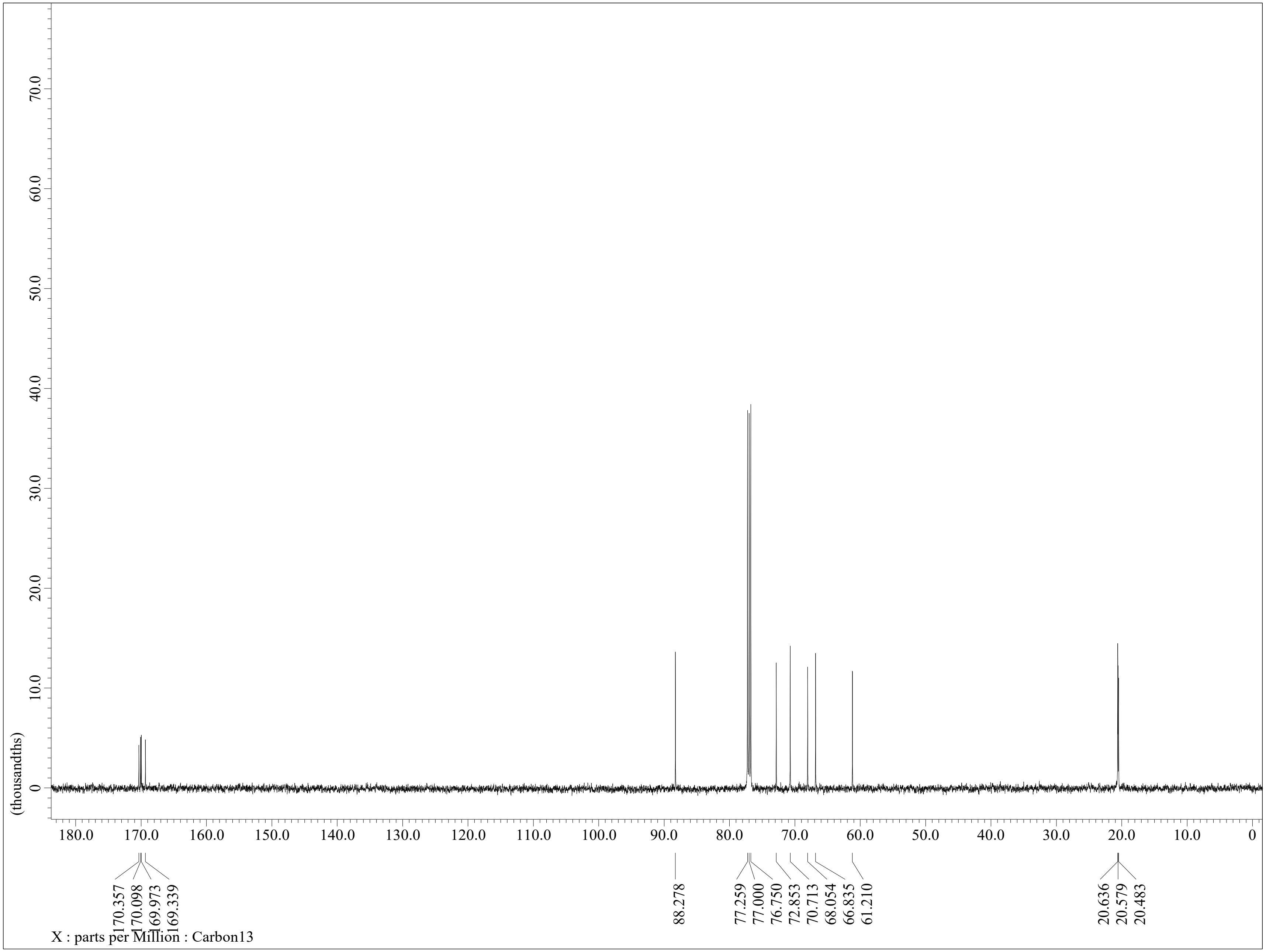

Spectra 2: ^{13}C NMR (125 MHz, $CDCl_3$) of compound **2**

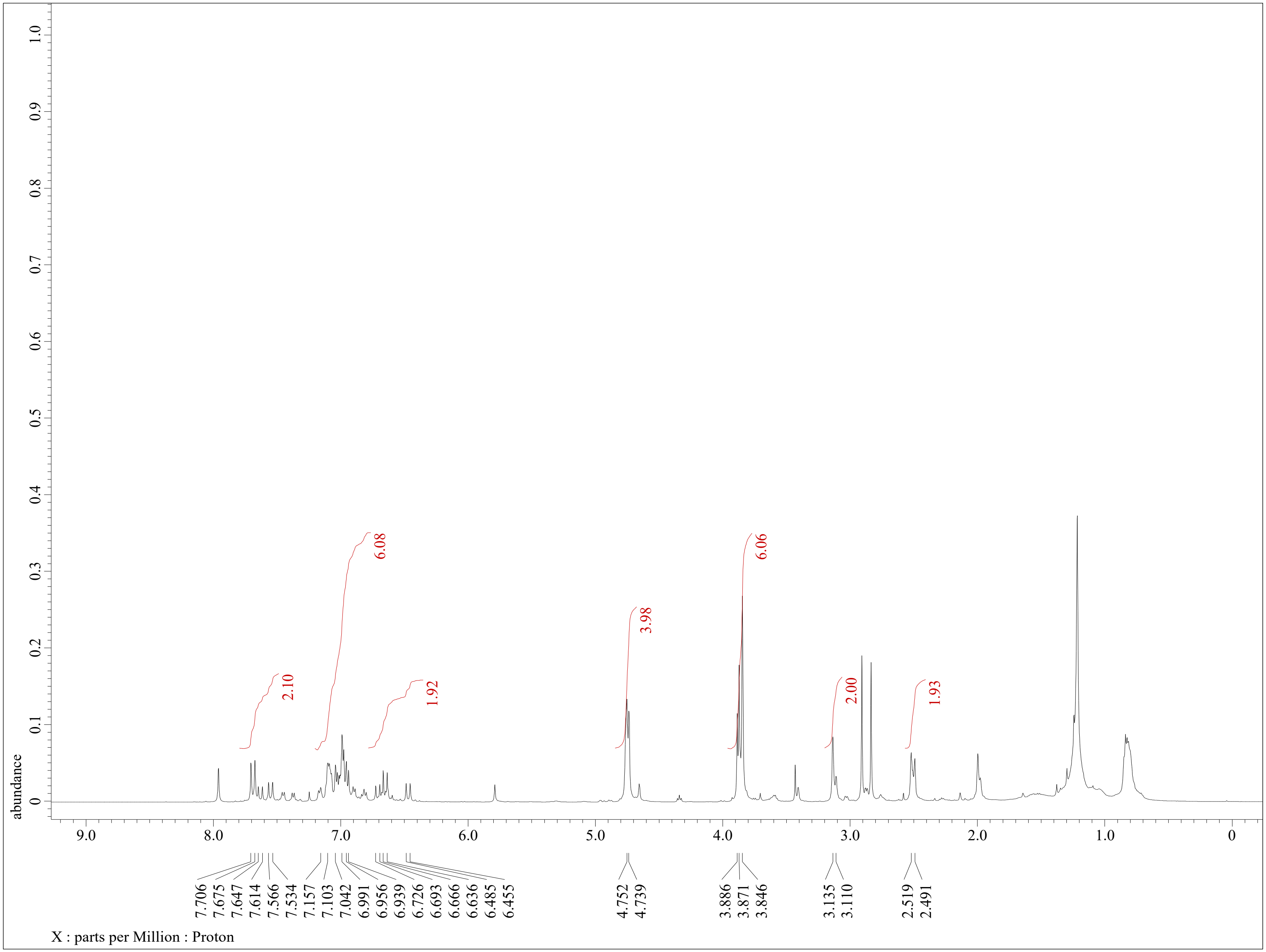

Spectra 3: ^{1}H NMR (500 MHz, $CDCl_3$) of compound **4**

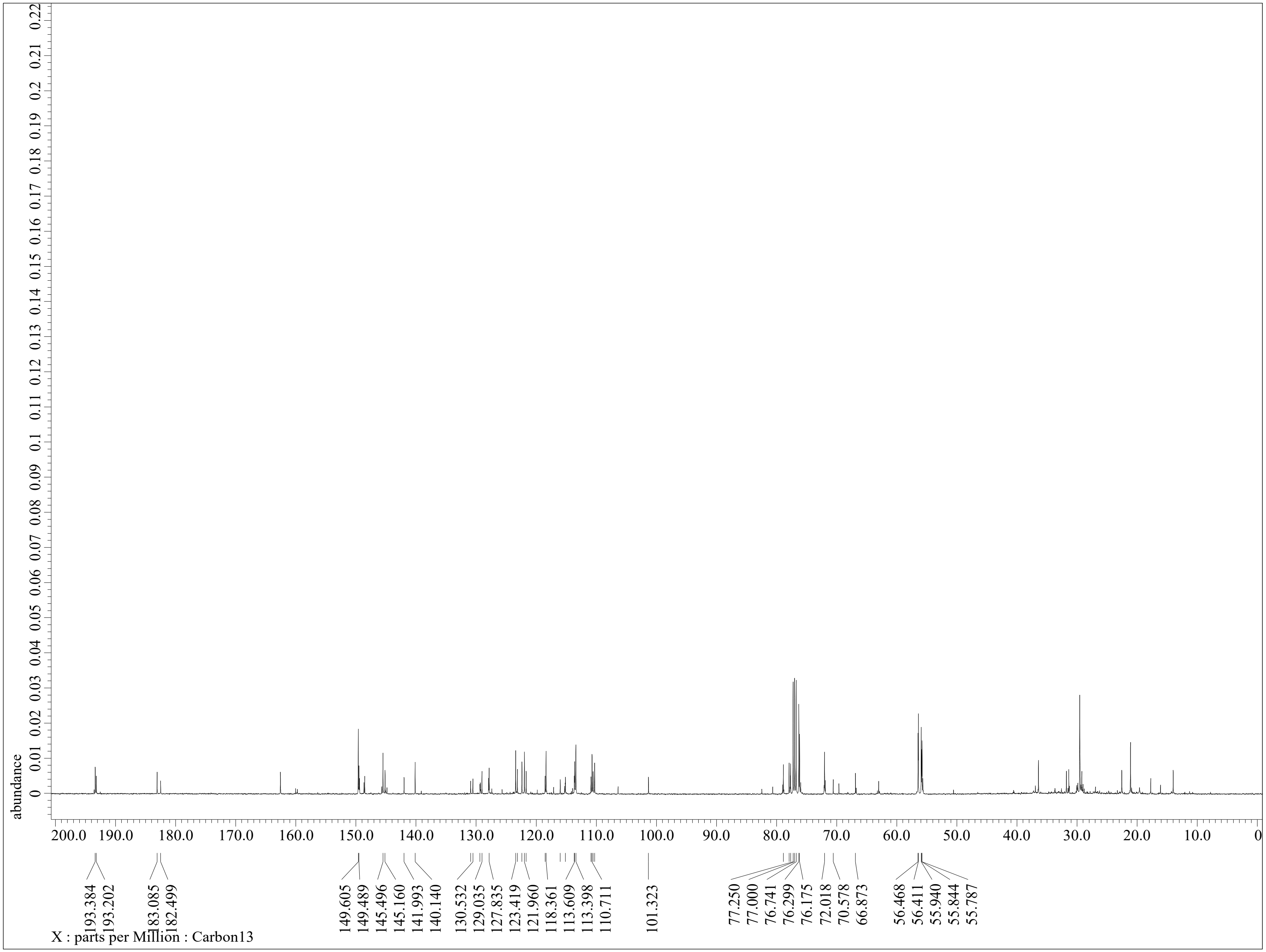

Spectra 4: ^{13}C NMR (125 MHz, $CDCl_3$) of compound **4**

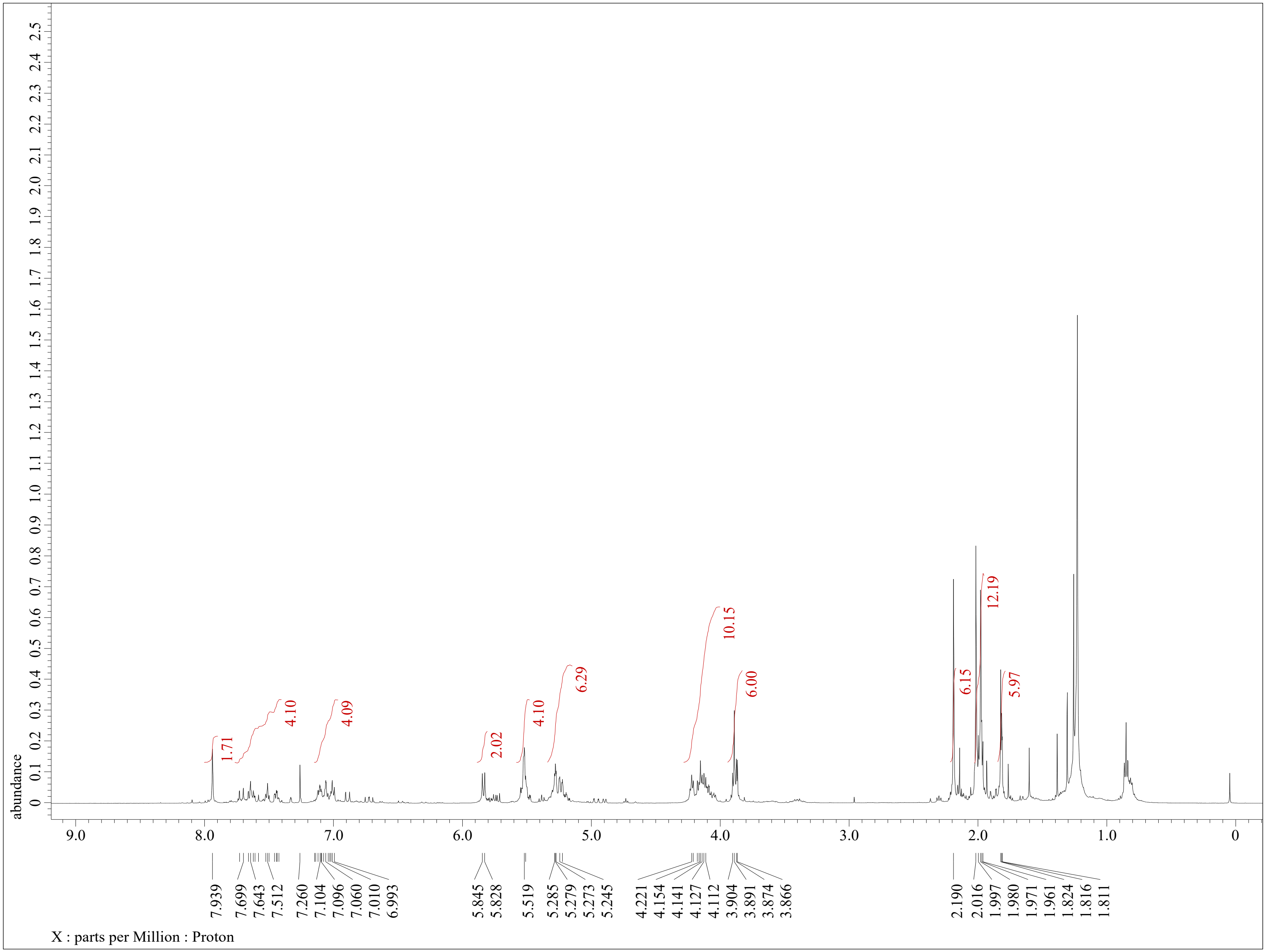

Spectra 5: ^{1}H NMR (500 MHz, $CDCl_3$) of compound **5**

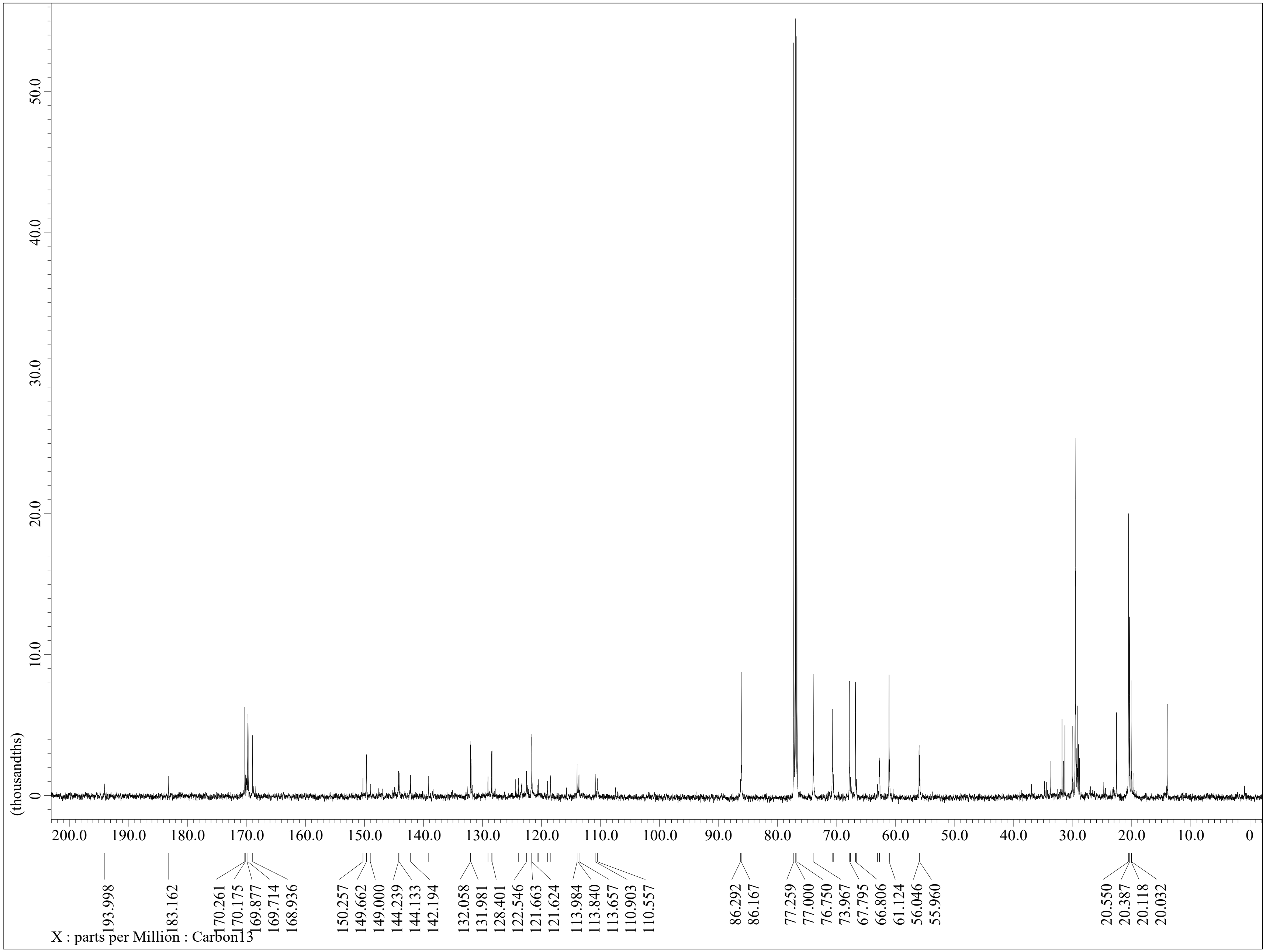

Spectra 6: ^{13}C NMR (125 MHz, $CDCl_3$) of compound **5**

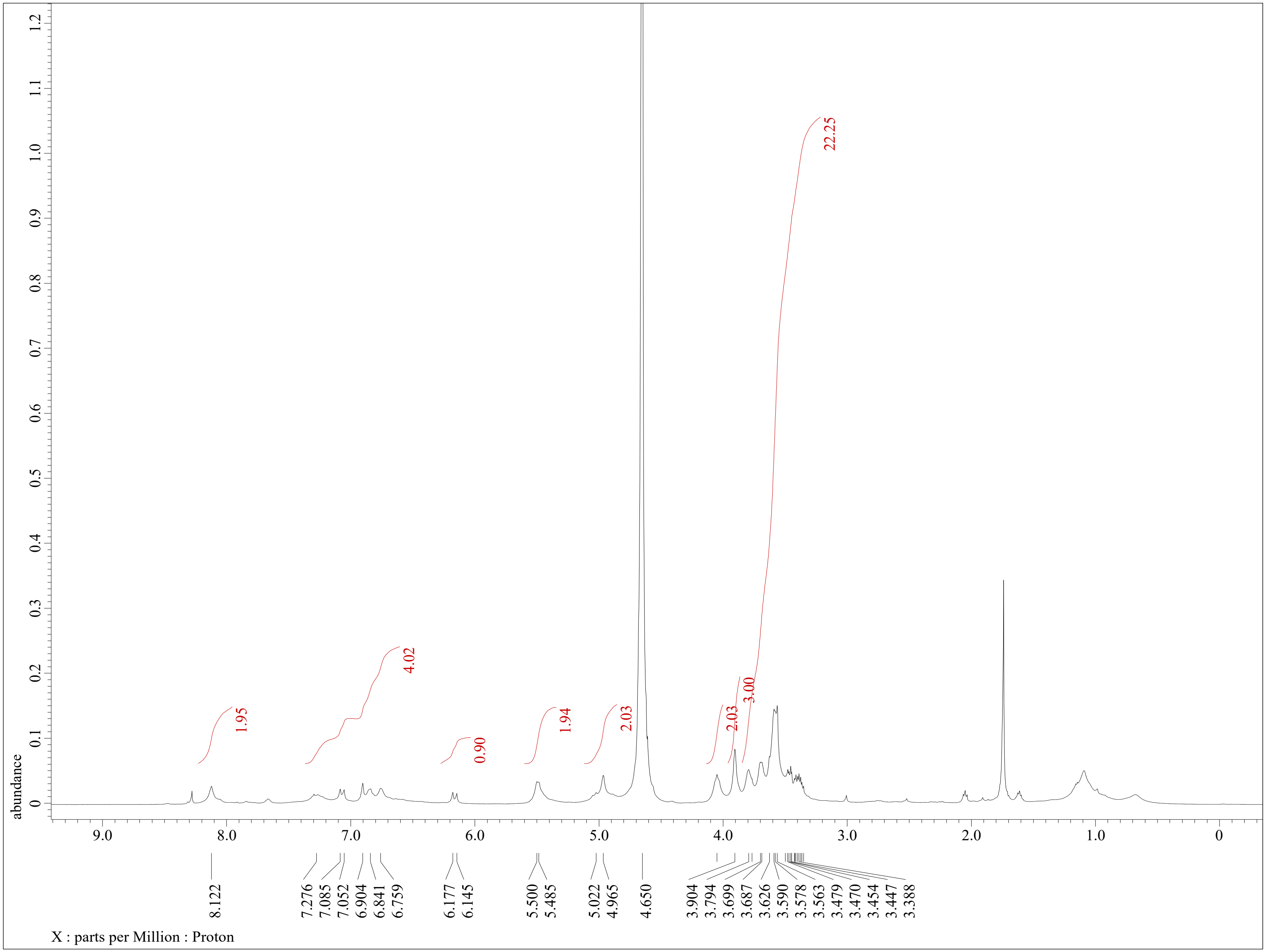

Spectra 7: ^{1}H NMR (500 MHz, D_2O) of compound **6**

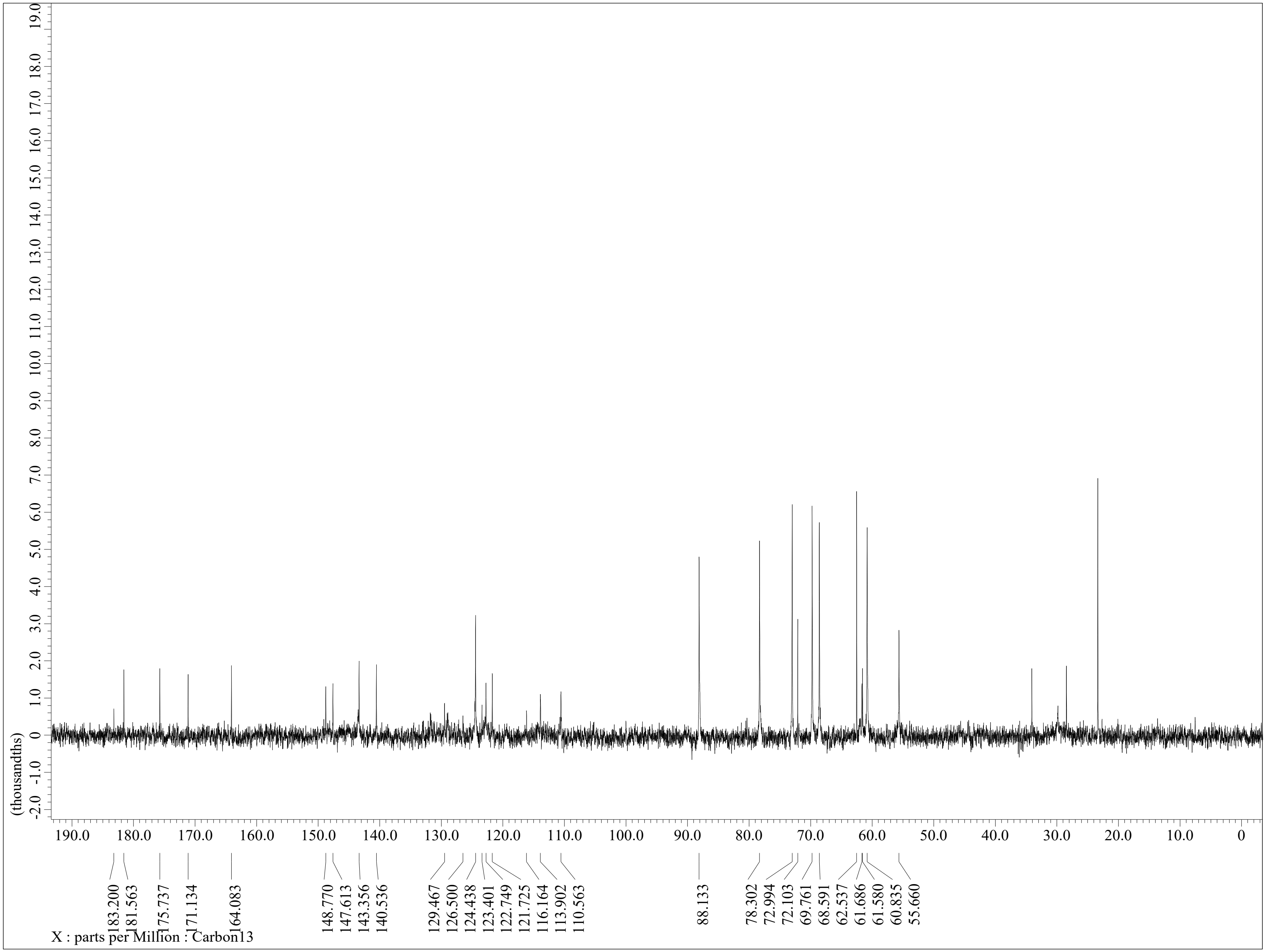

Spectra 8: ^{13}C NMR (125 MHz, D_2O) of compound **6**

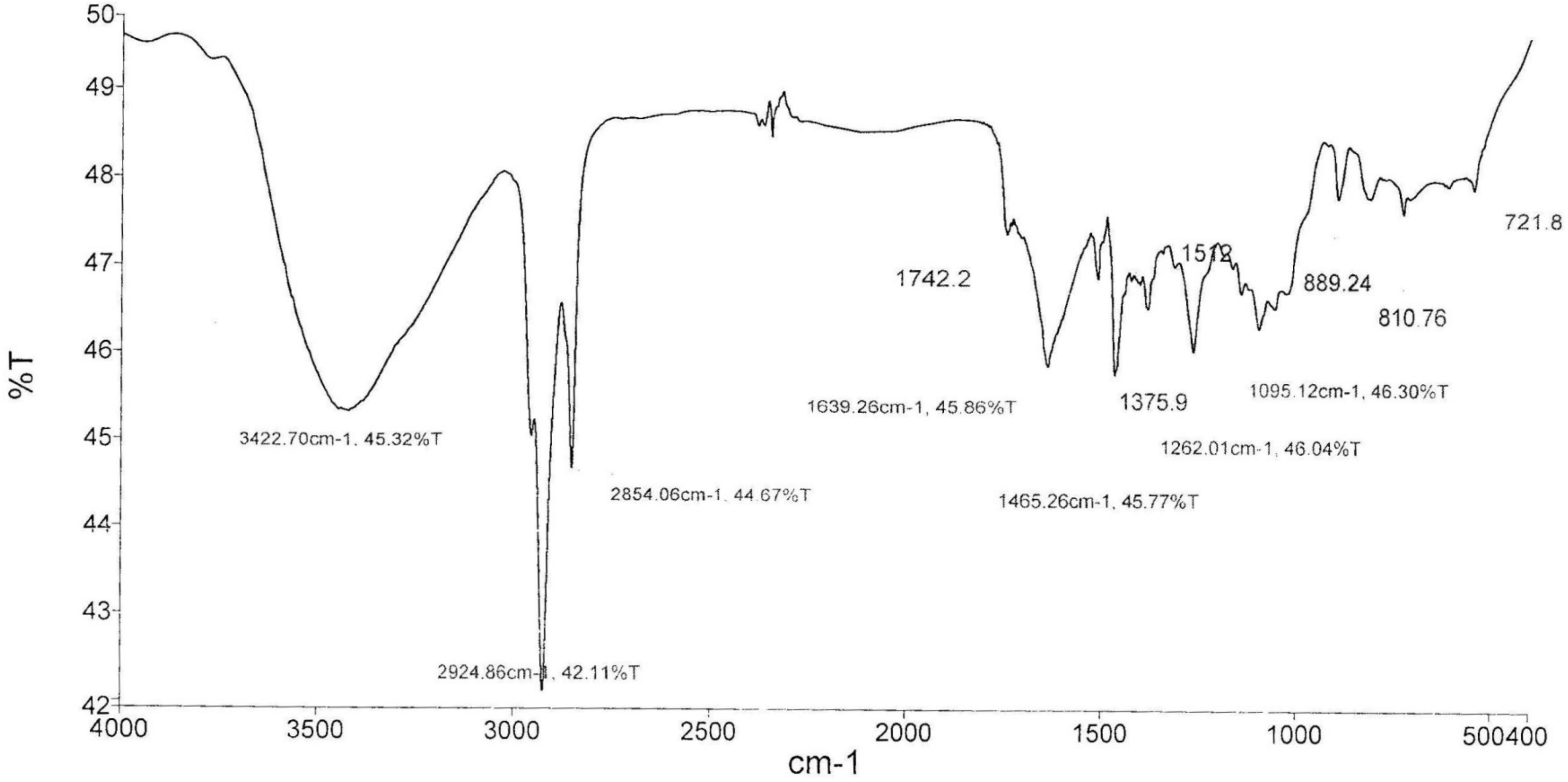

Spectra 9: IR Spectra of compound **5**

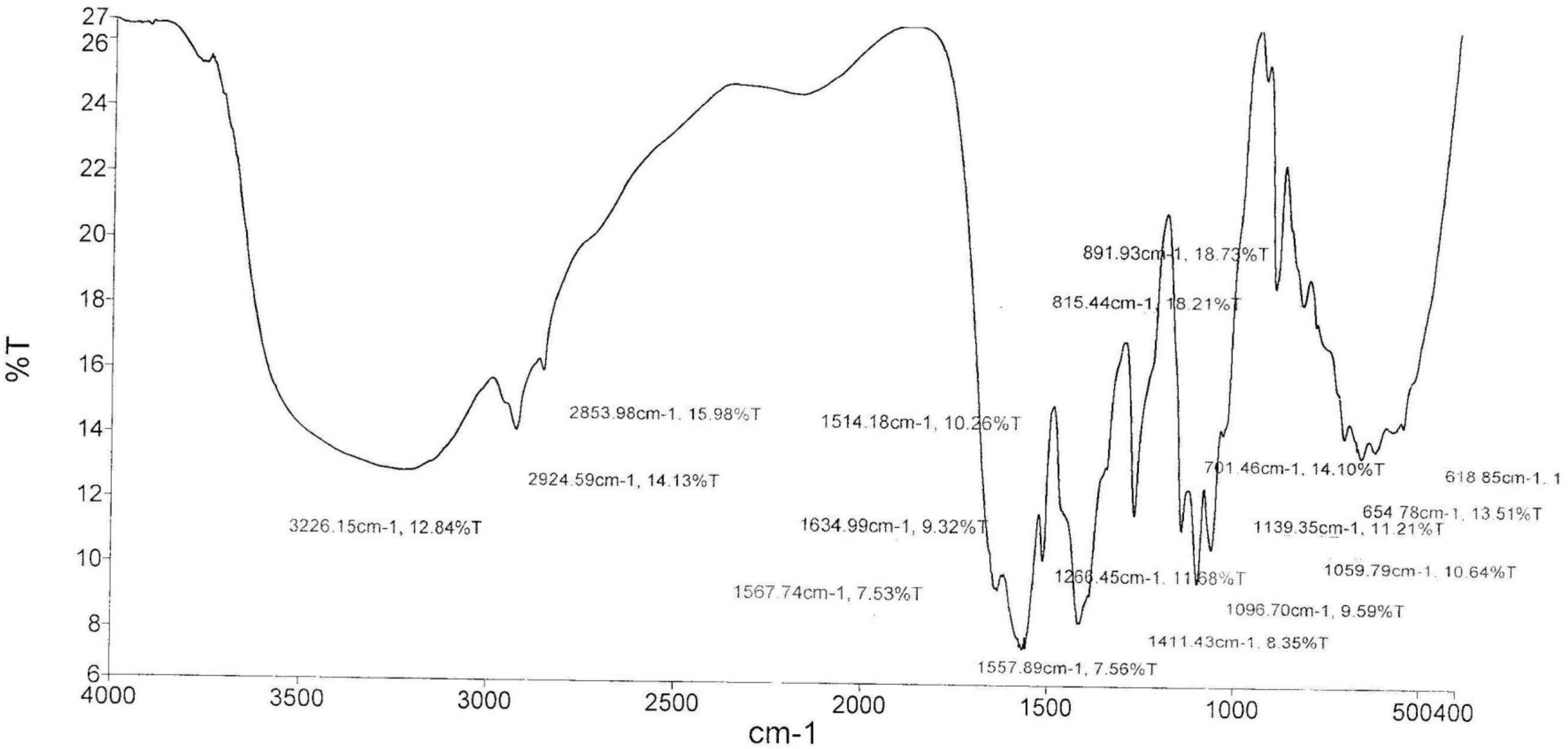

Spectra 10: IR Spectra of compound **6**

S3: The mCIM and eCIM results for clinical isolates of *K. pneumoniae*

Iso	Mer	mCIM	eCIM
1	<1	-ve	-ve
2	<1	-ve	-ve
3	<1	-ve	-ve
4	<1	-ve	-ve
5	<1	-ve	-ve
6	<1	-ve	-ve
7	<1	-ve	-ve
8	<1	-ve	-ve
9	<1	-ve	-ve
10	<1	-ve	-ve
11	<1	-ve	-ve
12	<1	-ve	-ve
13	<1	-ve	-ve
14	< 1	-ve	-ve
15	< 1	-ve	-ve
16	< 1	-ve	-ve
17	< 1	-ve	-ve
18	< 1	-ve	-ve
19	< 1	-ve	-ve
20	256	+ve	-ve
21	128	-ve	-ve
22	128	-ve	-ve
23	128	+ve	+ve
24	128	-ve	-ve

25	64	+ve	+ve
26	64	+ve	+ve
27	64	+ve	+ve
28	64	+ve	+ve
29	64	+ve	+ve
30	64	+ve	-ve
31	64	+ve	-ve
32	64	+ve	-ve
33	64	+ve	+ve
34	64	+ve	+ve
35	64	+ve	+ve
36	64	+ve	+ve
37	64	+ve	+ve
38	32	+ve	+ve
39	32	-ve	-ve
40	32	+ve	-ve
41	32	-ve	-ve
42	32	+ve	-ve
43	32	+ve	-ve
44	32	+ve	+ve
45	32	+ve	+ve
46	32	+ve	+ve
47	32	+ve	+ve
48	32	+ve	+ve
49	32	+ve	-ve
50	32	+ve	-ve

51	32	+ve	+ve
52	32	+ve	+ve
53	32	+ve	+ve
54	32	+ve	+ve
55	32	+ve	+ve
56	16	+ve	-ve
57	16	-ve	-ve
58	16	+ve	+ve
59	16	+ve	+ve
60	16	+ve	+ve
61	16	+ve	+ve
62	16	+ve	+ve
63	16	+ve	-ve
64	16	+ve	-ve
65	16	+ve	-ve
66	16	+ve	-ve
67	16	+ve	+ve
68	16	+ve	+ve
69	16	+ve	+ve
70	16	+ve	-ve
71	16	+ve	+ve
72	16	+ve	+ve
73	16	+ve	+ve
74	8	+ve	+ve
75	8	+ve	+ve
76	8	+ve	-ve

77	8	+ve	-ve
78	8	+ve	+ve
79	8	+ve	+ve
80	8	+ve	+ve
81	8	+ve	+ve
82	8	+ve	+ve
83	8	+ve	+ve
84	8	+ve	-ve
85	8	+ve	-ve
86	8	+ve	+ve
87	4	+ve	-ve
88	4	+ve	-ve
89	4	+ve	-ve
90	4	-ve	-ve
91	4	-ve	-ve
92	4	-ve	-ve
93	4	-ve	-ve
94	2	-ve	-ve
95	2	-ve	-ve
96	1	-ve	-ve
97	1	-ve	-ve
98	4	-ve	-ve
99	16	-ve	-ve
100	16	-ve	-ve
101	16	-ve	-ve
102	16	+ve	+ve

103	16	+ve	+ve
104	16	+ve	+ve
105	16	+ve	+ve
106	16	+ve	+ve
107	8	+ve	+ve
108	32	-ve	-ve
109	32	-ve	-ve
110	32	-ve	-ve
111	32	-ve	-ve
112	32	-ve	-ve
113	32	-ve	-ve
114	32	-ve	-ve
115	32	+ve	+ve
116	32	+ve	+ve
117	32	+ve	+ve
118	32	+ve	+ve
119	32	+ve	+ve
120	128	-ve	-ve
121	128	-ve	-ve
122	64	-ve	-ve
123	64	+ve	+ve
124	64	+ve	+ve
125	64	-ve	-ve
126	64	-ve	-ve
127	64	-ve	-ve
128	64	+ve	+ve

129	64	-ve	-ve
130	64	-ve	-ve
131	16	-ve	-ve
132	8	+ve	-ve
133	8	-ve	-ve
134	8	+ve	-ve
135	8	+ve	-ve
136	8	+ve	-ve
137	8	+ve	-ve
138	8	+ve	+ve
139	8	+ve	+ve
140	8	+ve	-ve
141	8	+ve	-ve
142	8	-ve	-ve
143	8	-ve	-ve
144	8	+ve	-ve
145	32	-ve	-ve
146	32	-ve	-ve
147	32	-ve	-ve
148	32	+ve	+ve
149	16	+ve	-ve
150	16	+ve	+ve
151	16	+ve	+ve
152	16	-ve	-ve
153	16	-ve	-ve
154	16	+ve	+ve

155	16	-ve	-ve
156	16	+ve	+ve
157	32	+ve	+ve

S4: The minimum inhibitory concentration (µg/ml) of the in vogue antibiotics along with protonophore, efflux pump inhibitor, proton pump inhibitor, and soluble curcumin against clinical isolates of *K. pneumoniae*

Iso	Mer	Mer/Sul	Imi	Ert	Ami	Gen	Lev	Cip	Cef	Cef/ Avi	CCCP	Vera	Vali	sCur
1	<1	2	2	<1	1024	32	<1	2	64	2	32	64	4	16
2	<1	<1	<1	<1	1024	1024	32	16	32	2	32	64	4	16
3	<1	<1	<1	<1	<1	<1	<1	<1	<1	<1	32	64	4	16
4	<1	<1	<1	<1	<1	<1	<1	<1	16	8	32	64	4	16
5	<1	<1	<1	<1	<1	<1	32	<1	4	4	32	64	4	16
6	<1	<1	<1	<1	<1	<1	<1	<1	<1	<1	32	64	4	16
7	<1	<1	<1	<1	2	<1	<1	<1	8	<1	32	64	4	16
8	<1	<1	<1	<1	>1024	1024	4	64	32	<1	32	64	4	16
9	<1	<1	<1	<1	<1	<1	<1	<1	4	<1	32	64	4	16
10	<1	<1	<1	<1	>1024	>1024	4	4	8	<1	32	64	4	16
11	<1	<1	<1	<1	<1	2	8	32	256	16	32	64	4	16
12	<1	<1	<1	<1	<1	<1	<1	<1	1	<1	32	64	4	16
13	<1	<1	<1	<1	128	16	<1	<1	4	<1	32	64	4	16
14	< 1	< 1	< 1	< 1	1024	32	32	8	64	2	32	64	4	16
15	< 1	2	< 1	< 1	2	1024	2	32	4	2	32	64	4	16
16	< 1	< 1	< 1	< 1	1024	1024	8	16	8	2	32	64	4	16
17	< 1	64	256	< 1	32	< 1	< 1	1	2	< 1	32	64	4	16
18	< 1	4	< 1	< 1	1024	64	32	1	16	2	32	64	4	16
19	< 1	< 1	< 1	< 1	< 1	<1	< 1	< 1	2	2	32	64	4	16
20	256	256	128	128	<1	<1	32	64	1024	64	32	512	64	64
21	128	64	128	64	1024	>1024	32	16	512	16	32	512	32	64
22	128	128	128	128	1024	1024	8	16	512	32	32	512	32	64
23	128	128	32	32	1024	1024	8	4	2	64	32	512	32	64

24	128	256	128	64	512	>1024	64	128	1024	256	32	512	32	64
25	64	64	32	32	1024	1028	16	16	256	32	32	256	16	64
26	64	64	8	64	1024	1028	4	16	512	32	32	256	16	64
27	64	64	32	64	1024	>1024	16	16	128	16	32	256	16	64
28	64	64	256	64	1024	>1024	16	64	512	32	32	256	16	64
29	64	64	16	32	1024	1024	64	16	512	8	32	256	16	64
30	64	128	32	32	< 1	< 1	128	32	16	2	32	256	16	64
31	64	128	64	64	1024	1024	8	16	512	16	32	256	16	64
32	64	64	32	64	>1024	1024	32	16	512	32	16	256	16	64
33	64	128	16	32	>1024	1024	32	128	512	8	16	256	16	64
34	64	256	32	64	1024	1024	16	16	1024	64	16	256	16	64
35	64	64	32	32	1024	1024	32	32	512	64	16	256	16	64
36	64	128	16	64	1024	1024	16	32	512	32	32	256	16	64
37	64	64	8	16	1024	1024	16	16	512	64	32	256	16	64
38	32	128	8	32	1024	1024	4	8	256	16	32	128	16	32
39	32	128	128	16	1024	1024	8	32	512	128	32	128	16	32
40	32	256	32	32	1024	1024	2	16	512	32	32	128	16	32
41	32	64	16	16	1024	1024	4	8	256	64	32	128	8	32
42	32	64	128	64	1024	>1024	16	16	256	128	32	128	8	32
43	32	64	128	64	1024	1024	32	16	512	32	32	128	8	32
44	32	32	64	128	1024	1024	32	256	128	16	32	128	8	32
45	32	64	16	32	1024	1024	8	64	256	32	32	128	8	32
46	32	64	16	32	1024	1024	16	64	512	16	32	128	8	32
47	32	32	64	32	>1024	>1024	64	64	512	16	32	128	8	32
48	32	64	8	16	1024	1024	16	32	512	64	32	64	16	32
49	32	64	8	16	1024	1024	8	16	1024	64	32	64	16	32

50	32	64	8	32	1024	1024	16	16	512	32	16	64	16	32
51	32	64	8	16	1024	1024	4	16	512	128	16	64	16	32
52	32	32	2	2	>1024	>1024	32	32	512	64	16	64	16	32
53	32	64	2	2	>1024	>1024	4	4	1024	32	16	64	16	32
54	32	64	8	2	>1024	>1024	16	128	1024	16	16	64	16	64
55	32	64	16	32	>1024	>1024	4	16	1024	32	16	64	16	64
56	16	64	16	16	1024	1024	8	8	128	16	16	128	16	32
57	16	16	16	8	1024	1024	16	16	256	2	16	128	16	32
58	16	128	16	8	1024	1024	4	16	512	128	16	128	16	32
59	16	16	32	8	1024	>1024	32	32	256	16	16	128	8	32
60	16	16	128	64	1024	1024	16	2	256	2	16	128	8	32
61	16	32	64	8	1024	1024	32	16	8	2	16	128	8	32
62	16	8	16	8	1024	>1024	32	8	64	64	16	128	8	32
63	16	64	16	4	1024	1024	32	16	16	4	32	128	8	32
64	16	4	4	4	>1024	>1024	32	128	32	<1	32	128	8	32
65	16	16	16	8	8	<1	16	64	512	16	32	128	4	32
66	16	32	64	16	1024	1024	4	8	512	32	32	128	4	32
67	16	32	8	4	1024	1024	4	16	512	128	32	128	4	32
68	16	16	8	4	1024	1024	8	32	128	<1	32	128	4	32
69	16	16	16	8	>1024	>1024	4	4	256	32	32	128	4	32
70	16	32	32	16	>1024	>1024	8	4	256	16	32	128	4	32
71	16	32	32	16	>1024	>1024	4	8	256	128	32	128	4	32
72	16	32	16	16	>1024	>1024	4	4	512	32	32	128	4	32
73	16	32	16	8	>1024	>1024	8	16	512	32	32	128	4	32
74	8	8	< 1	< 1	< 1	< 1	2	8	128	2	32	64	4	16
75	8	8	2	2	256	64	32	32	4	2	32	64	4	16

76	8	8	8	4	2	<1	16	256	4	2	32	64	4	16
77	8	32	32	8	1024	1024	32	16	512	8	32	64	4	16
78	8	8	2	< 1	1024	1024	4	32	32	2	32	64	4	16
79	8	16	4	4	>1024	>1024	64	64	512	32	32	64	4	16
80	8	8	32	16	2	<1	2	8	32	<1	32	64	2	16
81	8	16	8	8	1024	1024	16	16	32	4	32	64	2	16
82	8	8	4	4	1024	1024	8	8	256	16	32	64	2	16
83	8	16	32	16	1024	1024	4	8	512	128	32	64	2	16
84	8	32	4	2	>1024	>1024	4	16	512	64	32	64	2	16
85	8	32	4	2	<1	<1	4	16	16	2	32	64	2	16
86	8	8	8	4	>1024	>1024	2	4	512	32	32	64	2	16
87	4	8	2	< 1	< 1	< 1	4	16	32	2	32	64	4	16
88	4	8	< 1	< 1	< 1	< 1	4	8	16	2	32	64	4	16
89	4	< 1	< 1	< 1	< 1	< 1	4	< 1	8	2	32	64	4	16
90	4	8	4	4	<1	<1	4	8	8	2	32	32	4	16
91	4	4	8	4	1024	1024	8	64	4	<1	32	32	4	16
92	4	16	16	8	<1	<1	4	16	16	<1	32	32	4	16
93	4	8	<1	<1	>1024	>1024	8	32	64	4	16	32	2	16
94	2	1	<1	<1	1024	>1024	32	16	16	2	16	32	2	16
95	2	4	8	8	1024	1024	16	64	16	2	16	32	2	16
96	1	1	2	<1	1024	1024	32	< 1	16	2	16	32	2	16
97	1	2	<1	<1	4	<1	<1	1	64	<1	32	32	1	16
98	4	8	4	4	<1	<1	4	8	8	2	32	32	1	16
99	16	32	64	16	1024	1024	4	8	512	32	32	32	4	16
100	16	32	8	4	1024	1024	4	16	512	128	32	32	4	16
101	16	16	8	8	1024	1024	8	32	128	<1	32	32	4	16

102	16	16	16	16	>1024	>1024	4	4	256	32	32	32	4	16
103	16	32	32	64	>1024	>1024	8	4	256	16	32	32	4	16
104	16	32	32	32	>1024	>1024	4	8	256	128	32	32	4	16
105	16	32	16	16	>1024	>1024	4	4	512	32	32	32	2	16
106	16	32	16	8	>1024	>1024	8	16	512	32	32	32	2	16
107	8	8	< 1	< 1	< 1	< 1	2	8	128	2	32	32	2	16
108	32	32	64	32	1024	1024	32	256	128	16	32	128	8	32
109	32	64	16	4	1024	1024	8	64	256	32	32	128	8	32
110	32	64	16	16	1024	1024	16	64	512	16	32	128	8	32
111	32	32	64	8	>1024	>1024	64	64	512	16	32	128	8	32
112	32	64	8	8	1024	1024	16	32	512	64	32	128	8	32
113	32	64	8	16	1024	1024	8	16	1024	64	32	128	8	32
114	32	64	8	8	1024	1024	16	16	512	32	32	128	8	32
115	32	64	8	16	1024	1024	4	16	512	128	32	128	8	32
116	32	32	2	< 1	>1024	>1024	32	32	512	64	32	128	8	32
117	32	64	2	< 1	>1024	>1024	4	4	1024	32	32	128	8	32
118	32	34	8	< 1	>1024	>1024	16	128	1024	16	32	128	8	32
119	32	64	16	32	>1024	>1024	4	16	1024	32	16	128	8	32
120	128	128	32	32	1024	1024	8	4	2	64	16	512	64	64
121	128	256	128	64	512	>1024	64	128	1024	256	16	512	64	64
122	64	64	32	32	1024	1028	16	16	256	32	16	128	64	64
123	64	64	8	8	1024	1028	4	16	512	32	16	128	16	64
124	64	64	32	16	1024	>1024	16	16	128	16	16	128	16	64
125	64	64	256	256	1024	>1024	16	64	512	32	16	128	16	64
126	64	64	16	32	1024	1024	64	16	512	8	32	128	16	64
127	64	128	32	32	< 1	< 1	128	32	16	2	32	128	16	64

128	64	128	64	64	1024	1024	8	16	512	16	32	128	16	64
129	64	64	32	32	>1024	1024	32	16	512	32	32	128	16	64
130	64	128	16	32	>1024	1024	32	128	512	8	32	128	16	16
131	16	32	16	16	>1024	>1024	8	16	512	32	32	64	16	16
132	8	8	< 1	< 1	< 1	< 1	2	8	128	2	32	64	4	16
133	8	8	2	< 1	256	64	32	32	4	2	32	64	4	16
134	8	8	8	8	2	<1	16	256	4	2	32	64	2	16
135	8	32	32	32	1024	1024	32	16	512	8	32	64	2	16
136	8	8	2	1	1024	1024	4	32	32	2	32	64	2	16
137	8	16	4	8	>1024	>1024	64	64	512	32	32	64	2	16
138	8	8	32	32	2	<1	2	8	32	<1	32	64	2	16
139	8	16	8	1	1024	1024	16	16	32	4	32	64	4	16
140	8	8	4	2	1024	1024	8	8	256	16	32	64	4	16
141	8	16	32	16	1024	1024	4	8	512	128	32	64	4	16
142	8	32	4	8	>1024	>1024	4	16	512	64	16	64	8	16
143	8	32	4	4	<1	<1	4	16	16	2	16	64	4	16
144	8	8	8	8	>1024	>1024	2	4	512	32	16	64	4	32
145	32	32	2	8	>1024	>1024	32	32	512	64	16	128	16	32
146	32	64	2	4	>1024	>1024	4	4	1024	32	16	128	16	32
147	32	64	8	16	>1024	>1024	16	128	1024	16	16	128	16	32
148	32	64	16	4	>1024	>1024	4	16	1024	32	16	128	16	32
149	16	64	16	16	1024	1024	8	8	128	16	32	128	16	32
150	16	16	16	16	1024	1024	16	16	256	2	32	128	16	32
151	16	128	16	32	1024	1024	4	16	512	128	32	128	16	32
152	16	16	32	32	1024	>1024	32	32	256	16	32	128	16	32
153	16	16	128	64	1024	1024	16	2	256	2	32	128	4	32

154	16	32	64	16	1024	1024	32	16	8	2	32	128	4	32
155	16	8	16	8	1024	>1024	32	8	64	64	32	128	4	32
156	16	16	32	32	1024	>1024	32	32	256	16	32	128	4	32
157	32	32	2	8	>1024	>1024	32	32	512	64	32	128	16	64

S5: The Antibiogram of the clinical isolates of *K. pneumoniae*

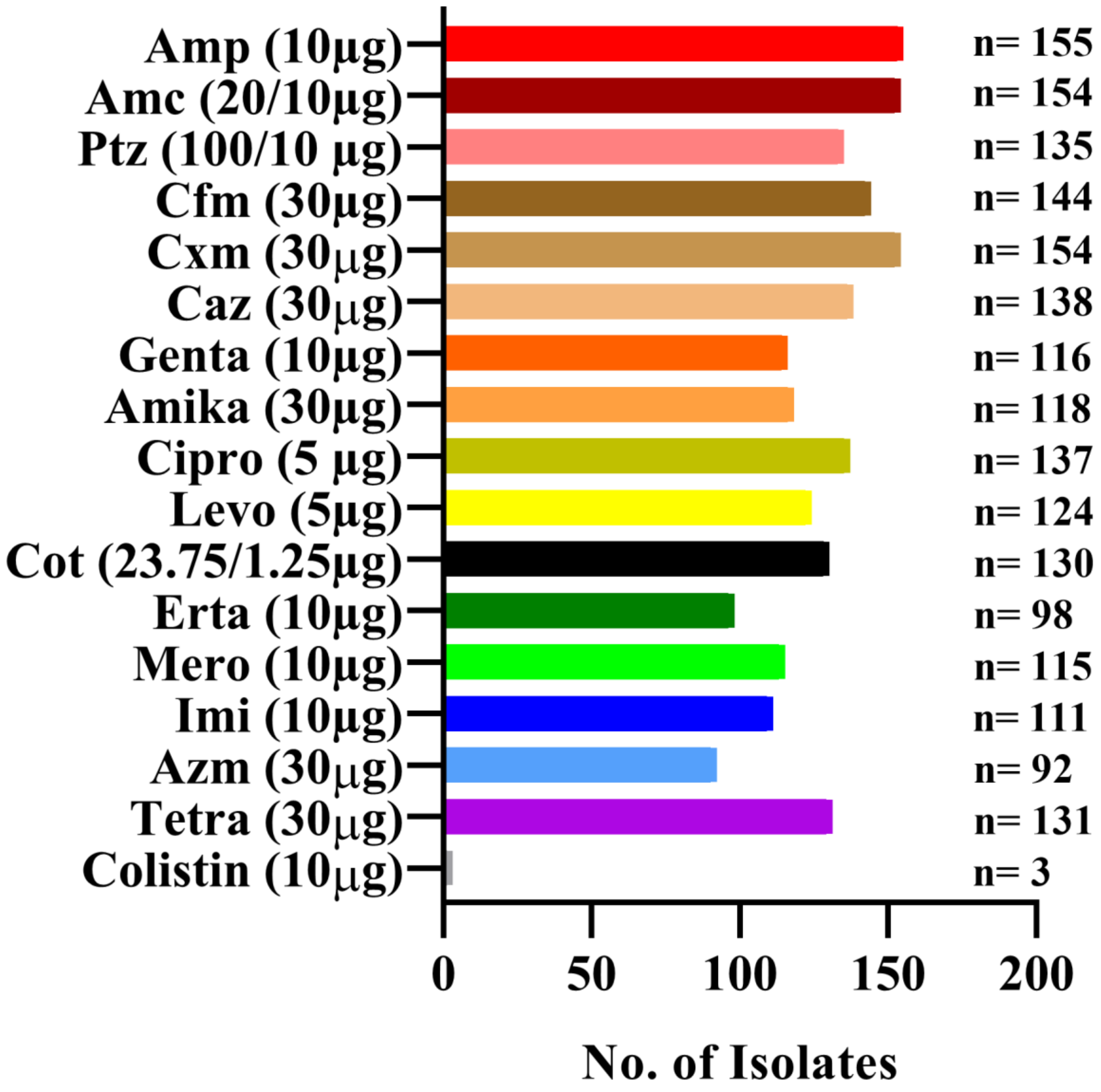

S6: Dendrogram of isolated isolates after ERIC PCR

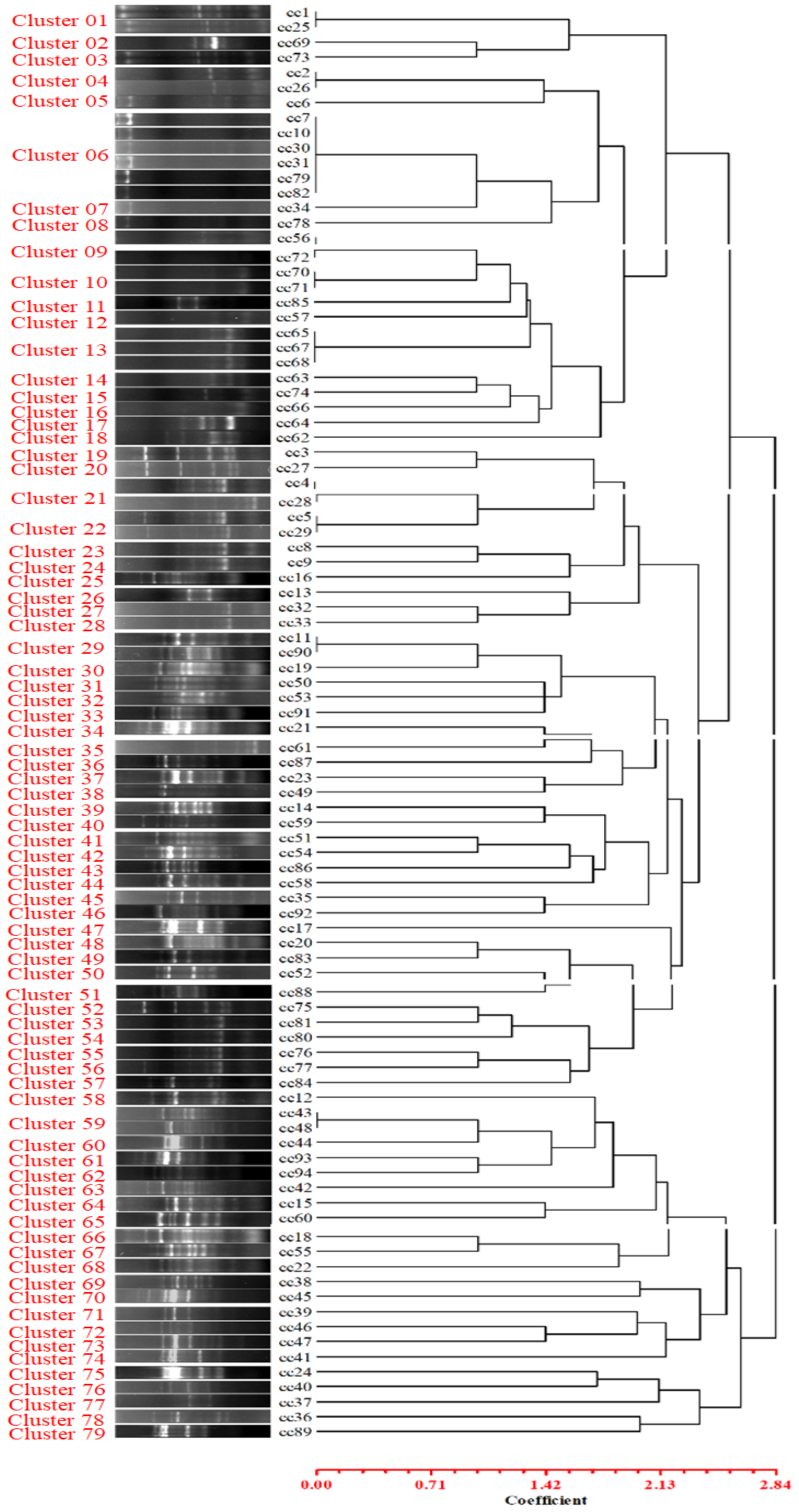

S7: Gating and area selection in Flow cytometry in electric potential assay.

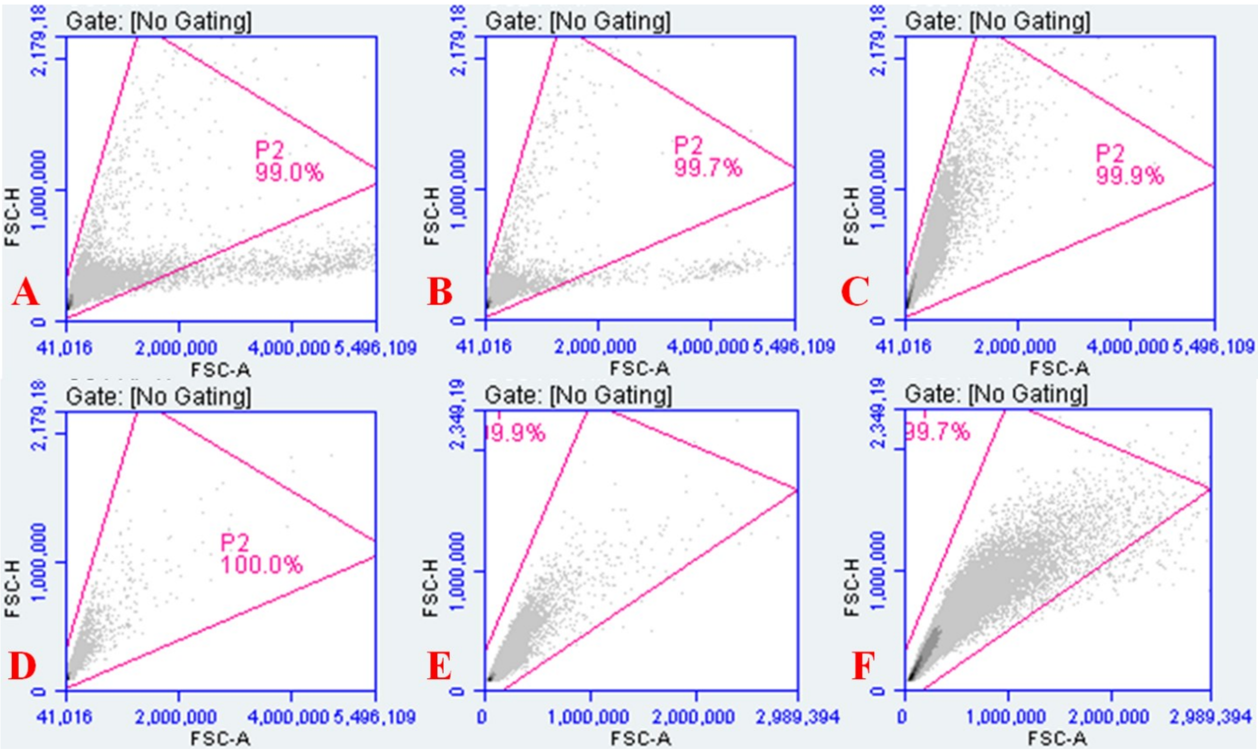

S8: Gating and area selection in Flow cytometry in membrane depolarization assay.

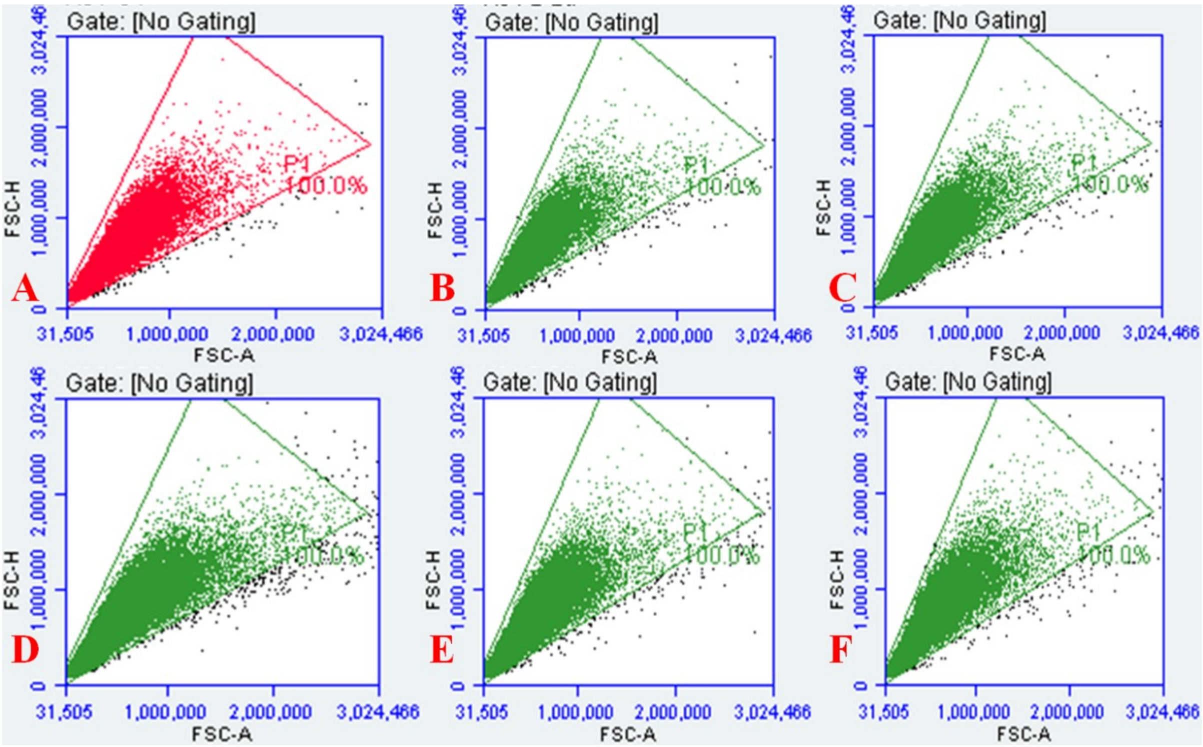

Supplement: Supplementary file 1 [file antibiotics-10-00388-s001.pdf]
